# Modeling Contraception and Pregnancy in Malawi: A *Thanzi La Onse* Mathematical Modeling Study

**DOI:** 10.1111/sifp.12255

**Published:** 2023-12-21

**Authors:** Tim Colbourn, Eva Janoušková, Ines Li Lin, Joseph Collins, Emilia Connolly, Matt Graham, Britta Jewel, Fannie Kachale, Tara Mangal, Gerald Manthalu, Joseph Mfutso‐Bengo, Emmanuel Mnjowe, Sakshi Mohan, Margherita Molaro, Wingston Ng'ambi, Dominic Nkhoma, Paul Revill, Bingling She, Robert Manning Smith, Pakwanja Twea, Asif Tamuri, Andrew Phillips, Timothy B. Hallett

## Abstract

Malawi has high unmet need for contraception with a costed national plan to increase contraception use. Estimating how such investments might impact future population size in Malawi can help policymakers understand effects and value of policies to increase contraception uptake. We developed a new model of contraception and pregnancy using individual‐level data capturing complexities of contraception initiation, switching, discontinuation, and failure by contraception method, accounting for differences by individual characteristics. We modeled contraception scale‐up via a population campaign to increase initiation of contraception (Pop) and a postpartum family planning intervention (PPFP). We calibrated the model without new interventions to the UN World Population Prospects 2019 medium variant projection of births for Malawi. Without interventions Malawi's population passes 60 million in 2084; with Pop and PPFP interventions. it peaks below 35 million by 2100. We compare contraception coverage and costs, by method, with and without interventions, from 2023 to 2050. We estimate investments in contraception scale‐up correspond to only 0.9 percent of total health expenditure per capita though could result in dramatic reductions of current pressures of very rapid population growth on health services, schools, land, and society, helping Malawi achieve national and global health and development goals.

## INTRODUCTION

Malawi has a high unmet need for contraception: 19 percent of married women who want to use contraception are not (National Statistics Office (NSO) [Malawi] and ICF 2017); and in total 42 percent of married women and 57 percent of unmarried sexually active women of reproductive age do not use contraception. Unplanned pregnancies are at higher risk for adverse outcomes for mothers and babies including maternal depression, and possibly stillbirth (Hall et al. 2018) and also, via unsafe abortion, maternal mortality (Polis et al. 2017). Low rates of contraception use also mean fertility remains high in Malawi at 4.2 children per woman (National Statistical Office (NSO) 2021). Consequently, the dependency ratio—the number of dependents (children aged 0–14 and adults aged over 65) per adult aged 15–64—remains high at an estimated 0.84 in 2020 (United Nations Department of Economic and Social Affairs Population Division 2019). This holds back development by reducing the resources available for each child's education and reducing the productive time of adults with additional dependent children (Cardona et al. 2020).

Although there is still a high unmet need for contraception and the associated problems highlighted above, access to and use of contraception in Malawi has improved dramatically since 1992 when only 7 percent of married women were using contraception, and 2004 when only 28 percent were using contraception (National Statistical Office (NSO) [Malawi] and ICF 2017).

We sought to develop a new model of contraception and pregnancy using individual‐level data that captures the complexities of contraception initiation, switching, discontinuation, and failure by contraception method and that can account for differences in these probabilities by age and other characteristics of the woman. This model was developed as a module within a new integrated epidemiological and economic model of all major health conditions and associated healthcare across the life‐course in Malawi which we call the *Thanzi La Onse* (TLO) model (The TLOmodel Team 2023). As part of the wider *Thanzi La Onse* model, our model of contraception and pregnancy aims to estimate the impact of contraception use changes on maternal, neonatal, and child mortality and morbidity and, indeed, all‐cause morbidity and mortality across the life‐course. Current models of contraception, including those used by international agencies such as Adding It Up, Impact 2, ImpactNow, Reality Check, and FamPlan/LIST, include unintended pregnancies and maternal mortality outcomes (Askew et al. 2017). However, they do not model morbidity, or outcomes over the life‐course, and require further work estimating causal effects on neonatal, infant, and child mortality (Askew et al. 2017). Furthermore, these models do not consider individual contraception journeys over reproductive ages including discontinuation and switching of methods, because they are not individual‐based simulation models. Our model, therefore, represents a step change in the ability to estimate changes in contraception use on health outcomes over the life‐course.

In this paper, we detail the model of contraception and pregnancy we have developed, its calibration and projections of resource use, pregnancies, births, and population size. The model incorporates trends and projections of increasing contraception use, and additional interventions to increase contraception uptake further. We focus on the impact on population size and the costs of increases in contraception coverage called for in the Malawi Costed Implementation Plan for Family Planning, 2016–2020 (CIP) (Government of Malawi 2015).

## METHODS

**FIGURE 1:**
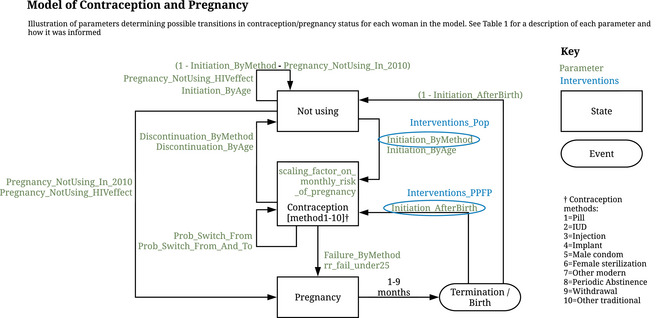
Conceptual overview of the model of contraception and pregnancy

The methods section is structured as follows. In the first subsection, our mathematical model of contraception and pregnancy (Figure 1) is explained in terms of its parameters and associated data sources and calculations (Table 1). Properties for each simulated woman, stored for use by the model parameters, and for analysis of model outputs, are shown in Table 2. This model—the contraception module—is linked to other modules within the overall *Thanzi La Onse* model (The TLOmodel Team 2023), and the source code is available from The TLOmodel Team (2023). In the second subsection, we describe modeling of contraception scale‐up by two interventions: population‐scope campaigns to increase contraception use (Pop) and postpartum family planning (PPFP). We include estimates of health system resources required for the use of each contraception method (Table 3) so that resource use can be estimated alongside contraception, pregnancy, and associated health outcomes. The final subsections detail our analyses and outcomes and ethics. The analyses are focused on assessment of effects of Pop and PPFP interventions to further increase contraception uptake. Changes in the number of births and population size assuming the introduction of interventions in 2023 compared to the UN World Population Prospects (WPP) 2019 medium variant projection, to which the model is calibrated, are predicted up to 2100. Trends in contraception uptake among women of reproductive age, calibrated to targets for each contraceptive method in the Malawi Costed Implementation Plan for Family Planning, 2016–2020 (CIP) (Government of Malawi 2015), are estimated for 2023–2050. As a consequence of increases in contraception uptake, decreases in the proportion of women of reproductive age becoming pregnant are estimated. We analyze costs for both the implementation of interventions to increase contraception uptake and the consequent increasing use of consumables associated with each contraceptive method, assuming the consumables are always available.

**TABLE 1:** Contraception and pregnancy model parameters

Parameter	Description	Value	Calculated from
Method_Use_In_2010	Proportion of women using each method in 2010, by age.	Table A1.2 in online Appendix 1	From DHS 2010 contraception calendar data (The Demographic and Health Surveys Program 2018), for each year of age of the woman from 15 to 49, the proportion of women who are using each of the 10 contraception methods (co_contraception, Table 2).
Pregnancy_NotUsing_In_2010	Probability per year of a woman not on contraceptive becoming pregnant, by age.	Table A1.2 in online Appendix 1	From Malawi DHS 2010 data (National Statistical Office (NSO) and ICF Macro 2011) using the data on births in the last year for each woman, and estimates of the relative risk of pregnancy given each contraceptive method and the proportion of women using each contraceptive method, as explained in online Appendix 1.
Pregnancy_NotUsing_HIVeffect	Relative probability of becoming pregnant while not using a contraceptive for HIV‐positive women compared to HIV‐negative women.	15–19: 1.4 20–24: 0.9 25–29: 0.8 30–34: 0.7 35–39: 0.5 40–44: 0.4 45–49: 0.3	From Marston, Zaba, and Eaton 2017 (Marston, Zaba, and Eaton 2017) Figure 1(a), age‐specific fertility rates (by five‐year age group: 15–19, 20–24, 25–29, 30–34, 35–39, 40–44, 45–49.
Age_specific_fertility_rates	Age‐specific fertility rates used for scheduling births in the first 9 months of the simulation. This is necessary because at the initiation of the simulation no women are pregnant.	15–19: 0.144 20–24: 0.239 25–29: 0.213 30–34: 0.174 35–39: 0.123 40–44: 0.061 45–49: 0.022	Data table from official source (United Nations World Population Prospects (United Nations Department of Economic and Social Affairs Population Division 2019)) for age‐specific fertility rates (by five‐year age group: 15–19, 20–24, 25–29, 30–34, 35–39, 40–44, 45–49) and calendar period (2010‐2014).
Scaling_factor_on_monthly_risk_of_pregnancy	Scaling factor (by five‐year age group: 15–19, 20–24, 25–29, 30–34, 35–39, 40–44, 45–49) on the monthly risk of pregnancy and contraceptive failure rate.	15–19: 1.227 20–24: 0.799 25–29: 0.829 30–34: 0.809 35–39: 0.749 40–44: 0.645 45–49: 0.941	Calibrated so that, at the beginning of the simulation, the age‐specific monthly probability of a woman having a live birth matches the United Nations World Population Prospects age‐specific fertility rate value for the same year. Calibration is done in two stages (explained in detail in online Appendix 1): 1. The scaling_factor_on_monthly_risk_of_pregnancy is used to induce the correct number of age‐specific births initially, given the initial pattern of contraceptive use. 2. Trends over time in the risk of starting (time_age_trend_in_initiation) and stopping (time_age_trend_in_stopping) contraception, adjusted for older women, are used to induce the correct trend in the number of live births over time.
Initiation_ByMethod	Probability per month of a woman who is not using any contraceptive method of starting use of a method, by method.	Table A2.1 in online Appendix 2	This was calculated from an analysis of DHS contraception calendar data (The Demographic and Health Surveys Program 2018) for Malawi DHS 2010 (National Statistical Office (NSO) and ICF Macro 2011), in Stata, as explained in online Appendix 2.
Initiation_AfterBirth	The probability of a woman starting a contraceptive immediately after birth, by method.	Table A2.2 in online Appendix 2	This was calculated from an analysis of DHS contraception calendar data (The Demographic and Health Surveys Program 2018) for Malawi DHS 2010 (National Statistical Office (NSO) and ICF Macro 2011), in Stata, as explained in online Appendix 2.
Prob_Switch_From	The probability per month that a woman switches from one method of contraceptive to another is conditional that she will not discontinue use of the method.	Table A3.1 in online Appendix 3	This was calculated from an analysis of Malawi DHS 2016 (National Statistical Office (NSO) [Malawi] and ICF 2017) contraception calendar data (The Demographic and Health Surveys Program 2018), in Stata, via competing risks regression, as explained in online Appendix 3.
Prob_Switch_From_And_To	The probability of switching to a new method, by method, conditional that the woman will switch to a new method.	Table A3.2 in online Appendix 3	This was calculated from an analysis of Malawi DHS 2016 (National Statistical Office (NSO) [Malawi] and ICF 2017) contraception calendar data (The Demographic and Health Surveys Program 2018), in Stata, as explained in online Appendix 3.
Failure_ByMethod	Probability per month of a woman on a contraceptive becoming pregnant, by method.	Table A3.3 in online Appendix 3	These parameters were calculated from an analysis of Malawi DHS 2016 (National Statistical Office (NSO) [Malawi] and ICF 2017) ^a^ contraception calendar data (The Demographic and Health Surveys Program 2018), in Stata, via a competing risks regression model (separate to that for switching), as explained in online Appendix 3.
Discontinuation_ByMethod	The probability per month of discontinuing use of a method, by method.	Table A3.4 in online Appendix 3	
rr_fail_under25	The relative risk of becoming pregnant while using a contraceptive for women younger than 25 years compared to older women.	2.2	From Guttmacher analysis (Polis et al. 2016) Table 9, page 52; see online Appendix 4 Table A4.1.^b^
Initiation_ByAge	The effect of age on the probability of starting use of contraceptive (add one for multiplicative effect).	Table A4.2 in online Appendix 4	This was calculated from an analysis of Malawi DHS 2016 (National Statistical Office (NSO) [Malawi] and ICF 2017) contraception calendar data (The Demographic and Health Surveys Program 2018), in Stata, using fractional polynomial regression (better fitting model, higher F statistic), as explained in online Appendix 4. The results of this model are plotted in online Figure A4.1 and are used to calculate the proportionate difference in Initiation_ByMethod probability from the average probability for each age in years. ^c^
Discontinuation_ByAge	The effect of age on the probability of discontinuing use of contraceptives (add one for multiplicative effect).	Table A4.3 in online Appendix 4	This was calculated from an analysis of Malawi DHS 2016 (National Statistical Office (NSO) [Malawi] and ICF 2017) contraception calendar data (The Demographic and Health Surveys Program 2018), in Stata, using fractional polynomial regression, as explained in online Appendix 4. The results of this model are plotted in online Figure A4.2 and are used to calculate the proportionate difference in Discontinuation_ByMethod probability from the average probability for each age in years.
Interventions_Pop	Pop (population scale contraception intervention) intervention multiplier of Initiation_ByMethod. Representing the method‐specific proportional increases due to Pop intervention.	Table A5.1 in online Appendix 5	Calibrated to meet the expected changes in the percentage of women using each method from our 2010 baseline to 2020 as a result of the Malawi Costed Implementation Plan For Family Planning 2016–2020 (CIP), estimated on pages 37–38 (Figure 31) of the CIP report (Government of Malawi 2015). Explained in detail in online Appendix 5.
Interventions_PPFP	PPFP (postpartum family planning) intervention multiplier of Initiation_AfterBirth. Representing the method‐specific proportional increases due to PPFP intervention.	Table A5.1 in online Appendix 5	

^a^ We use DHS 2016 data only because the reason for discontinuation (failure) is not in the DHS 2010 contraception calendar data.

^b^ Other “lifestyle” variables potentially associated with increased probabilities of failure (marital status, parity, wealth, urban‐rural, education) were not included because the effect estimates for these were not statistically significant for >50 percent of those using contraception and only significant for one or two minor contraception categories–see Table A4.1— in Appendix 4.

^c^ We note a similar analysis was done for Initiation_AfterBirth though the model did not result in statistically significant or large enough effect estimates to be considered important enough to include. A simpler model with age and age‐squared was also not significant, and a very simple model with just age resulted in the Initiation_AfterBirth probabilities only changing by ∼±10–15 percent throughout the 15–49 age range (*p* = 0.03), that is, not an important enough change to make it worth adding an additional parameter (Initiation_AfterBirth_ByAge), especially given Initiation_AfterBirth is much rarer than initiation (Initation_ByMethod).

**TABLE 2:** Properties of each individual in the contraception and pregnancy model simulation

Property	Description	Categories
co_contraception	“Current contraceptive method” (categorical variable with 11 categories)	“not_using,” “pill,” “IUD,” “injection,” “implant,” “male_condom,” “female_sterilization,” “other_modern,” “periodic_abstinence,” “withdrawal,” “other_traditional”^a^
is_pregnant	Whether individual is currently pregnant	True, False
date_of_last_pregnancy	Date that the most recent or current pregnancy began.	DATE
co_unintended_preg	Whether the most recent or current pregnancy was unintended.	True, False
co_date_of_last_fp_appt	The date of the most recent family planning appointment. This is used to determine if a family planning appointment is needed to maintain the person on their current contraceptive. If the person is to maintain use of the current contraceptive, an appointment will be scheduled only if the days elapsed since this value exceeds the method‐specific parameter days_between_appts_for_maintenance (see right‐hand column)	DATE set for each method via days_between_appts_for_maintenance: IUD: 4,383 days, implant: 1,461 days injections: 91 days male_condom: 91 days other_modern (female condom^a^): 91 days, pill: 91 days

co_ is used as a prefix for properties of the contraception module

^a^ These are the 11 categories of contraception (“not using” + 10 methods) from the DHS analysis of initiation, discontinuation, failure, and switching probabilities. “other modern” includes male sterilization, female condom, emergency contraception (in Malawi only female condom is used by more than a very few people so we only include female_condom in this category). “other traditional” includes lactational amenorrhoea, standard days method, “other traditional method.”

**TABLE 3:** Health system resources: Consumables

Contraception package	Item	Expected units per case	Unit cost 2021 price in MWK	Unit cost 2021 price in USD^a^
Contraception initiation	Pregnancy Slide Test Kit (Human ‐ Chorionic Gonadotrophin (Hcg))_100_MM192300_CMST, strip	1	32	0.04
pill	Ethinylestradiol 0.03 mg + levonorgestrel 0.15 mg_Each_FP000800_CMST, cycle //80% patients//; or Microlut (Levonorgestrel 0.03 mg)_Each_FP004200_CMST, cycle //20% patients//	3.75	493^b^	0.62
IUD	Glove disposable powdered latex medium_100_HH077700_CMST, pair	2	37	0.05
	IUD, Copper T‐380A	1	26	0.03
Injections	Medroxyprogesterone acetate injection 150 mg/mL, 1 mL vial with 2 mL syringe with 22 g 0.7 × 25 mm needle_Each_BB049500_CMST, ampule	1	481	0.61
	Glove disposable powdered latex medium_100_HH077700_CMST, pair	1	37	0.05
	Water for injections, 10ml_Each_BB077100_CMST	1	32	0.04
	Povidone iodine 10% solution_200 ml_DN004470_CMST, 5 mL sachet	1	85	0.11
	Gauze, swabs 8‐ply 10cm x 10cm_100_FF010800_CMST	1	16	0.02
Implant	Gauze, swabs 8‐ply 10cm x 10cm_100_FF010800_CMST	1	16	0.02
	Jadelle(implant)_Each_FP003700_CMST, 2 rods //50% patients//; or Implanon (Etonogestrel 68 mg)_Each_FP004100_CMST, 1 rod //50% patients//	1	760^c^	0.96
	Trocar	0.1	312	0.39
	Syringe, needle + swab	2	200	0.25
	Needle suture intestinal round bodied ½ circle trocar_6_CMST	1	179	0.23
	Lidocaine HCl (in dextrose 7.5%), ampoule 2 mL	2	310	0.39
	Glove disposable powdered latex medium_100_HH077700_CMST, pair	3	37	0.05
	Povidone iodine 10% solution_200ml_DN004470_CMST, 5 mL sachet	1	85	0.11
Male condom	Condom, male	30	21	0.03
Female sterilization	Povidone iodine 10% solution_200ml_DN004470_CMST, 5 mL sachet	2	85	0.11
	Paracetamol 500 mg, tablets_1000_AA049500_CMST, tablet	8	4	0.01
	Glove surgeons size 7 sterile_Pair_HH080400_CMST	2	302	0.38
	Tape, adhesive, 2.5 cm wide, zinc oxide, 5 m roll	0.25	1558	1.97
	Catgut chromic suture sterile 0, 75 cm, round‐bodied ½ circle 40 mm needle_12_GG000600_CMST	3	307	0.39
	Gauze, swabs 8‐ply 10cm x 10cm_100_FF010800_CMST	2	16	0.02
	Syringe, autodestruct, 5ml, disposable, hypoluer with 21g needle_Each_HH150000_CMST + alcohol swabs/wipes 70% isopropyl alcohol 100 pieces_100_FF000300_CMST	3	154	0.19
	Diazepam 5 mg/mL, 2ml_Each_BB024000_CMST	1	130	0.17
	Atropine sulphate 600 micrograms/ml, 1ml_Each_BB006600_CMST //1 unit 50% patients → 0.5 units per case//	0.5	121	0.15
	Lidocaine HCl (in dextrose 7.5%), ampoule 2 mL	1	310	0.39
	Cotton wool, 500g_Each_FF007800_CMST	0.2	2690	3.4
	Polyamide monofilament suture sterile 1, on 40 mm 3/8 circle reverse cutting needle_12_GG005100_CMST	3	179	0.23
other modern	Female Condom_Each_FP003500_CMST	30	22	0.03

^a^ 1 USD = 790 MWK, xe.com:771–818 MWK in 2021: https://www.xe.com/currencycharts/?from=USD&to=MWK&view=5

^b^ Weighted average cost of the alternatives. According to the Essential Health Package 2021 data assembled to cost the Health Sector Strategic Plan III (2023–2030) (Health Benefits Package data) (Government of the Republic of Malawi 2023), Levonorgestrel 0.15 mg + ethinyl estradiol 30 mcg (Microgynon) is given to 80 percent of patients and Levonorgestrel 0.0375 mg to 20 percent of patients. The cost per cycle is 531.1 MWK (2021) and 340.4 MWK (2021), respectively.

^c^ Weighted average cost of the alternatives. According to the Essential Health Package 2021 data assembled to cost the Health Sector Strategic Plan III (2023–2030) (Health Benefits Package data) (Government of the Republic of Malawi 2023), Jadelle and Implanon (Etonogestrel 68 mg) are given to 50% of patients. The unit cost per rod is 449.05 MWK and 622.12, respectively. Two rods of Jadelle, but only 1 rod of Implanon are used. Jadelle lasts five years, and Impanon lasts three years, therefore we assume the time between appointments to be four years (=0.5*5 + 0.5*3), which is 1,461 days (Table 2).

### Model of Contraception and Pregnancy

The *Thanzi La Onse* contraception module covers baseline contraception methods use (one of 10 methods or “not_using”; Figure 1), contraception initiation probabilities for each method by age, contraception failure (pregnancy) for each method by age, contraception initiation probabilities for each method after pregnancy, probabilities of switching between contraceptive methods, and discontinuation probabilities by age, as described in Figure 1. It also determines fertility via age‐specific fertility rates from the WPP (United Nations Department of Economic and Social Affairs Population Division 2019) and calibration to the WPP medium variant population projection for Malawi, as explained below. Descriptions of the parameters and properties of the contraception and pregnancy model, together with their sources, are provided in Tables 1 and 2 and the online Supporting Information. Each parameter is also explained briefly below. We used the Malawi Demographic and Health Survey (DHS) (National Statistical Office (NSO) and ICF Macro 2011; National Statistical Office (NSO) [Malawi] and ICF 2017) contraception calendar data to calculate the contraception initiation, switching, discontinuation, and failure probabilities by contraception method. We followed the guidance in the DHS contraception calendar tutorial (The Demographic and Health Surveys Program 2018) using Stata 15.1 statistical software (Stata Corp LP, College Station, TX, USA). We estimated the probabilities for 2010 as our baseline year and used a time step of one month. The model is coded in Python with all code and resources available on our website (The TLOmodel Team 2023).

In 2010, at the beginning of the simulation, women are assigned a contraception method, depending on their age, according to the proportion of women using each of the 10 methods, the Method_Use_In_2010 parameter (Table 1). In 2010, those not using contraception have a probability of becoming pregnant by age, represented by the Pregnancy_NotUsing_In_2010 parameter calculated from DHS data on births in the last year for each woman, relative risks of pregnancy given the use of each contraception method and the proportion of women using each method (Table 1 and online Appendix 1). This probability of pregnancy for women not using contraception is adjusted for HIV‐positive women via the Pregnancy_NotUsing_HIVeffect parameter, as per analysis of the relationship between HIV and fertility in the era of antiretroviral therapy in sub‐Saharan Africa by Marston, Zaba, and Eaton (2017) (Table 1).

The age_specific_fertility_rates (Table 1) are age‐specific fertility rates, by five‐year calendar period, for Malawi from the WPP (United Nations Department of Economic and Social Affairs Population Division 2019), used for scheduling births in the first nine months of the simulation. This is necessary because at the initiation of the simulation no women are pregnant. We use the scaling_factor_on_monthly_risk_of_pregnancy to induce the correct number of age‐specific pregnancies initially (and subsequent births), given the initial pattern of contraceptive use. Trends over time in the risk of starting and stopping contraception, adjusted for older women, are then used to induce the correct trend in the number of live births over time, that is, to calibrate to the WPP medium variant population projection (United Nations Department of Economic and Social Affairs Population Division 2019) for Malawi (Table 1 and online Appendix 1).

We calculated monthly initiation probabilities for each of the 10 contraception methods after a period of not using contraception, Initiation_ByMethod, or for the month following pregnancy/birth/termination, Initiation_AfterBirth (Table 1 and online Appendix 2). However, in order to not allow sterilization in women who are too young, the initiation probability of female sterilization in women younger than 30 years was set to zero. To preserve the overall probabilities, the probability of sterilization in older women was increased accordingly, and the probabilities of initiating other contraception methods in both age groups were adjusted.

We calculated monthly probabilities for switching from each of the 10 contraception methods, Prob_Switch_From, and probabilities of switching to another specific method determined by the Prob_Switch_From_And_To parameter (Table 1). Contraception switching was determined by a competing risks regression model of two risks: switching method and any other change in contraception status, explained in online Appendix 3. Monthly Failure_ByMethod and Discontinuation_ByMethod probabilities (Table 1) for each of the 10 contraception methods were calculated via a separate competing risks regression model of seven risks: contraception failure and discontinuation due to any of six reasons (“desire to become pregnant,” “other method related reason,” “side effects,” “wanted more effective method,” “other fertility‐related reasons,” “other reason/don't know”), also explained in online Appendix 3.

Following an analysis, by the Guttmacher Institute, of variables potentially associated with an increase in contraception failure in 10 East African countries (Polis et al. 2016), and considering the need for parsimony in our model we concluded that there was only sufficient evidence for an increase in contraception failure (relative risk of 2.2) for those aged under 25, rr_fail_under25 (Table A4.1 in online Appendix 4). Women under 25 had statistically significantly higher failure rates than those over 25 for the following methods of contraception: pill, injection, and periodic abstinence, and higher failure rates for almost all of the other contraception methods with data. In contrast, there was only a statistically significant difference in failure of male condoms by marital status, with “ever married” having a higher failure rate than “never married” and the direction of this relationship was the opposite, though not statistically significant, for other contraceptive methods (online Appendix 4 Table A4.1); and the same was true for parity: only a significant difference for injections, and there was an opposite, though not statistically significant, relationship for at least one of the other methods. We included proportional incremental changes in contraception initiation, Initiation_ByAge, and discontinuation, Discontinuation_ByAge, probabilities by age in years of the woman (Table 1), calculated using fractional polynomial regression, as explained in online Appendix 4.

### Contraception Interventions and Costs

We model two interventions, in addition to the status quo, based on the ambitious Malawi Costed Implementation Plan for Family Planning, 2016–2020 (CIP) (Government of Malawi 2015): (a) Pop and (b) PPFP. The intervention parameters are shown in Figure 1 and described in Table 1 and below. The Pop intervention is modeled as increased initiation probabilities of the modern contraception methods after not using any contraception by the Interventions_Pop multiplier of Initiation_ByMethod. The PPFP intervention is modeled as increased initiation probabilities after the end of pregnancy by the Interventions_PPFP multiplier of Initiation_AfterBirth. These multipliers are calibrated to target coverage for each contraception method defined in the CIP (Table 1 and online Appendix 5). The increases in initiation due to interventions are in addition to the expected increases in contraception use that mirror expected fertility decline and slowing population growth in Malawi according to the UN World Population Prospects (United Nations Department of Economic and Social Affairs Population Division 2019). The annual national cost estimates for implementing Pop and PPFP interventions for 2016, taken from the Malawi Costed Implementation Plan For Family Planning (Government of Malawi 2015), are provided in online Appendix 5 Table A5.1. The costs of the Pop intervention cover demand creation activities including information, education and communication outreach and social behavior change communication campaigns involving use of mass media, peer educators, community engagement, and engagement of key stakeholder groups including youth in and out of school, and religious, traditional, and community leaders (online Appendix 5) (Government of Malawi 2015). The costs of the PPFP intervention are for integration of contraception services into postpartum care (online Appendix 5) (Government of Malawi 2015). We calculate an approximate annual average of the costs across the five‐year period 2016–2020 assuming the increases and decreases in costs for each of the five years presented in the CIP reflect differences in start‐up and implementation costs. We assume these annual national costs for 2016 reflect the size of the population of women and men of reproductive age (15–49) covered by these interventions; hence, we adjust them for each year simulated with interventions proportionally to the increasing population estimated in the simulation.

We obtained the health system resources and their cost used as a package to deliver each method of contraception from the data assembled to cost the Health Sector Strategic Plan III (2023–2030) (Health Benefits Package data) (Government of the Republic of Malawi 2023). The amount of resources used for each method was matched with the database assembled for the One Health Tool (OHT) (World Health Organization 2012) (used to cost Malawi's Health Sector Strategic Plan II (2017–2022) (Government of the Republic of Malawi 2023), OHT 2016 data) whenever possible, for the remaining sources the amounts are taken from Malawi Health Benefits Package data. Costs are reported in 2021 Malawi Kwacha (MWK) and 2021 United States Dollars (USD, $) using an exchange rate of $1 = 790 MWK (Table 3). The costs of interventions are then calculated as the implementation costs of Pop and PPFP interventions (these are also reported in 2021 MWK), and increased costs of consumables due to increased intervention uptake.

### Analyses and Outcomes

**FIGURE 2:**
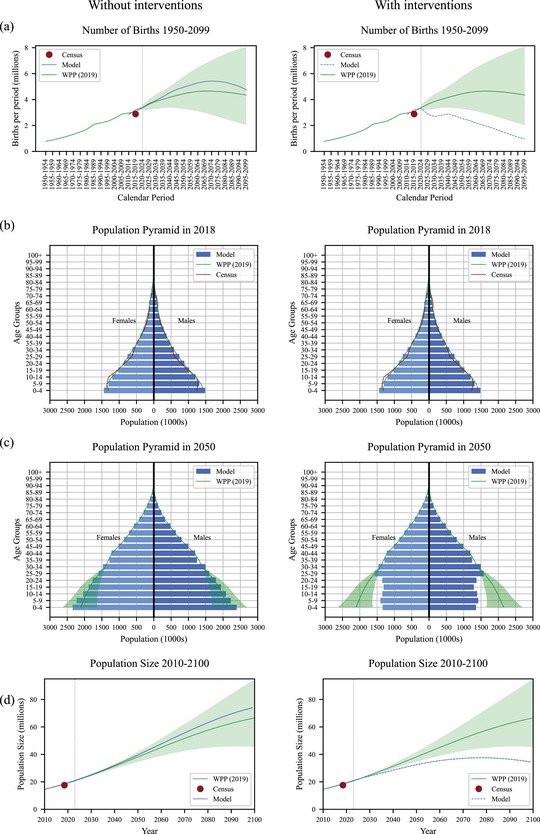
Model births and population results: (a) number of births, (b) population pyramid in 2018, (c) population pyramid in 2050, and (d) population size. (Left) Calibration (without Pop and PPFP interventions). (Right) Effect of Pop and PPFP interventions starting 2023. The dotted vertical line shows the start of the interventions, year 2023

**FIGURE 3:**
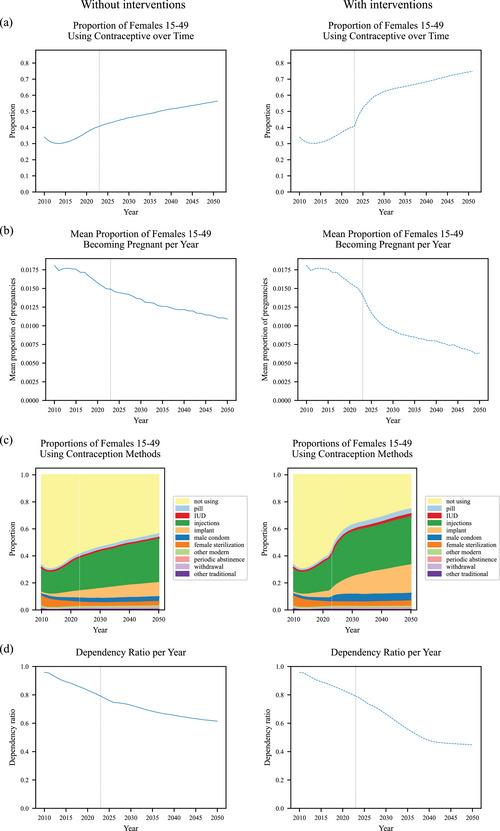
Model results without and with Pop and PPFP interventions: (a) proportion of females 15–49 years old using a contraceptive, (b) mean proportion of females 15–49 years old becoming pregnant per year, (c) proportions of females 15–49 years old using contraception methods, and (d) dependency ratio: number of dependents (children aged 0–14 and adults aged over 65) per adult aged 15–64. (Left) without Pop and PPFP interventions. (Right) with Pop and PPFP interventions since 2023. The dotted vertical line shows the start of the interventions, year 2023

**FIGURE 4:**
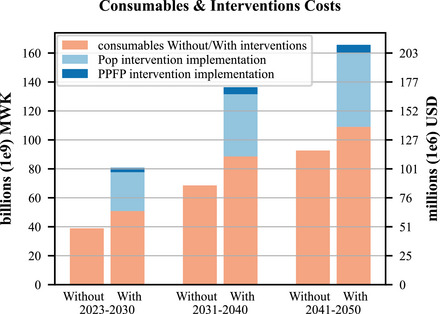
Contraception consumables and intervention costs with and without interventions 2023–2050

To calibrate our model, the scaling_factor_on_monthly_risk_of_pregnancy parameter is fitted so the population resulting from the pregnancies and births predicted by the parameters of the contraception model, without the Pop and PPFP interventions, from 2010 onwards is a good approximation of existing data on the population of Malawi from the 2018 census (National Statistical Office (NSO) 2019) and modeled estimates to 2100 from the medium variant of the UN World Population Prospects (United Nations Department of Economic and Social Affairs Population Division 2019) (more details are provided in online Appendix 1). Deaths are applied (by the Thanzi La Onse demography module (The TLOmodel Team 2023)) as per the WPP death schedule enabling comparison of projected total population sizes. As well as visually assessing the fit (Figure 2 left), we calculate the root mean squared deviance (RMSD) between the model and the WPP medium variant projection to assess the “fit” of the model as the percentage of RMSD between medium variant and high variant WPP projection. We then run the model with the Pop and PPFP interventions to see their effect (Figure 2 right). We plot contraception use (by method), pregnancies per year as mean proportions of women becoming pregnant among all women of reproductive age (15–49), and dependency ratio as projected by our model, both without and with interventions (Figure 3; the corresponding total numbers of women are shown in online Appendix 6 Figure A6.1). We also plot and summarize contraception use and costs (by method) within decades from intervention implementation in the model in 2023 up to 2050 without and with interventions (Figure 4 and Table 4). We run our model using a simulated population of 250,000 people representative of the Malawian population (The TLOmodel Team 2023) and scale results to the total population of Malawi. 250,000 people provide sufficient precision and variation of <0.2 percent in the percentage of all women using each contraceptive method across runs due to the use of random number generators governing which individuals in the simulation are selected for each change in contraception status in each time step. We, therefore, round percentages using each method to the nearest 0.1 percent and numbers of women using each method to the nearest 1000. We do not include uncertainty around our model parameter values so do not run more than one run or present uncertainty in our model outputs.

### Ethics

The *Thanzi La Onse* project received ethical approval from the College of Medicine Malawi Research Ethics Committee (COMREC, P.10/19/2820) in Malawi. Only anonymized secondary data are used in the *Thanzi La Onse* model including in the contraception model presented in this paper; therefore, individual informed consent was not required.

## RESULTS

Figure 2 (left) plots the calibration results for births (Figure 2a, left), population pyramid (Figures 2b and 2c, left), and total population (Figure 2d, left). Our base model, that is, without additional interventions, predicts the number of births in Malawi to the year 2100 expected by the UN World Population Prospects medium variant projection well (Figure 2a, left) with RMSD corresponding to 23 percent of RMSD between medium variant and high variant WPP projection. The model population size also closely matches the WPP medium variant projection to 2100—a population of over 60 million and still increasing by the end of the century (Figure 2d, left). The population pyramid produced by the model for 2018 is a close match to that of the WPP medium variant projection and the 2018 Malawi census (Figure 2b, left) as is the population pyramid produced by the model for 2050, which is within the WPP uncertainty interval (green‐shaded area, Figure 2c, left). Figure 2 (right) shows the effect of Pop and PPFP interventions starting in 2023. The births are reduced to below the WPP low variant projection (Figure 2a, right), the population pyramid becomes thinner at ages below 25 by 2050 (Figure 2c, far right), and the total population size is considerably reduced such that after reaching a high of around 35 million it starts to decline in the latter part of the century (Figure 2d, right).

Figure 3 plots the without and with intervention results in detail from 2010 to 2050 (Figure 3, left: no interventions; Figure 3, right: with interventions). Without Pop and PPFP, interventions contraception use increases steadily to 2050, with more women aged 15–49 using contraception than not using contraception from 2037 onwards (Figure 3a, left). The proportion of women becoming pregnant reflects this (Figure 3b, left). The most popular contraception method is injection (>30 percent of women aged 15–49 by 2030; >35 percent by 2050), followed by an implant, male condom, female sterilization, pill, and intrauterine device (IUD) (Figure 3c, left). The dependency ratio, representing pressure on the productive population, declines steadily from 0.96 in 2010 to 0.61 in 2050. With Pop and PPFP interventions starting in 2023, contraception use increases sharply with more women using contraception than not using contraception from mid‐2024 onwards, then increases more steeply than without interventions, through to 2050 (Figure 3a, right compared to Figure 3a, left). The proportion of women who become pregnant within a year declines rapidly from 2023 after Pop and PPFP interventions are started (Figure 3b, right). With Pop and PPFP interventions, the most popular method is also injection, followed by implant—with around half as many women using an implant as injection (Figure 3c, right) compared to less than one third without the Pop and PPFP interventions (Figure 3c, left), followed again by male condom, female sterilization, pill, and IUD (Figure 3c, right). The dependency ratio decline is more rapid from 0.96 in 2010 to 0.44 in 2050.

Table 4 and Figure 4 summarize contraception usage and resource costs (by method) without and with the Pop and PPFP interventions, for 2023–2030, 2031–2040, and 2041–2050. With interventions, the most popular method is injection (31 percent of women during 2023–2030, rising to 35 percent of women during 2041–2050), followed by implant (10 percent of women during 2023–2030, rising to 20 percent of women during 2041–2050, followed by male condom, female sterilization, and pill (Table 4). Female sterilization is the most expensive method per woman (Table 3) though few new female sterilizations take place as the method is permanent once performed so total costs for female sterilization over 2023–2050 are modest and injections and pills make up the largest fractions of the consumable costs, followed by male condoms and implants (Table 4). Pop and PPFP intervention costs together are around 54 percent of the consumable costs for all modern methods combined (Table 4). The PPFP intervention is a targeted intervention for postpartum women and costs only 11 percent of the population‐wide Pop intervention, so the vast majority of the combined Pop and PPFP intervention costs are for the Pop intervention (Table 4). Estimated implementation costs were 120.7 billion MWK (152.8 million USD) for the Pop intervention and 13.6 billion MWK (17.2 million USD) for the PPFP intervention and an additional 248.5 billion MWK (314.5 million USD) for associated contraceptive consumables over 2023–2050. In contrast, the estimated cost over 2023–2050 for existing trends of increasing contraception with no interventions was 200.1 billion MWK (253.3 million USD) for consumables (Table 4). For the estimated mean population of Malawi over 2023–2050 with contraception interventions of 26,948,785 (Figure 2d, right), the additional $231.2 million cost of the interventions over the 28‐year period from 2023 to 2050 (Table 4 and Figure 4) is $0.31 per capita per year, or 0.9 percent of the estimated $34.4 total health expenditure per capita per year averaged over the last five years of available data: 2016–2020 (latest available data; World Health Organization 2023).

**TABLE 4:** Contraception usage and resources costs by method without and with intervention—2023–2050

Contraception method^a^	2023–2030				2031–2040			
	Without interventions		With Pop and PPFP interventions		Without interventions		With Pop and PPFP interventions	
	Mean percentage of women using (thousands of users)	Costs in millions of MWK (thousands of USD)	Mean percentage of women using (thousands of users)	Costs^b^ in millions MWK (thousands USD)	Mean percentage of women using (thousands of users)	Costs in millions of MWK (thousands of USD)	Mean percentage of women using (thousands of users)	Costs^b^ in millions MWK (thousands USD)
Pill	2.1% (125)	6,089 (7,707)	3.1% (189)	9,294 (11,765)	2.3% (172)	10,458 (13,238)	3.5% (268)	16,272 (20,597)
IUD	0.4% (24)	4 (5)	1.1% (65)	17 (21)	0.5% (37)	6 (8)	1.8% (135)	25 (32)
Injections	26.6% (1,589)	26,815 (33,943)	31.1% (1,861)	31,509 (39,885)	29.5% (2,239)	47,091 (59,609)	33.4% (2,532)	53,149 (67,278)
Implant	6.3% (380)	2,111 (2,672)	9.6% (583)	3,563 (4,510)	8.4% (640)	4,268 (5,403)	15.9% (1,209)	8,083 (10,232)
Male condom	3.1% (186)	3,082 (3,902)	5.2% (310)	5,189 (6,568)	3.4% (256)	5,292 (6,699)	5.6% (427)	8,800 (11,140)
Female sterilization	3.0% (177)	587 (743)	3.4% (200)	784 (993)	2.8% (212)	1,003 (1,270)	3.4% (258)	1,157 (1,464)
other modern	0.2% (14)	235 (298)	0.5% (29)	507 (642)	0.3% (21)	451 (571)	0.7% (52)	1,113 (1,409)
Modern contraceptives TOTAL	41.7% (2,494)	38,922 (49,269)	53.9% (3,238)	50,863 (64,383)	47.1% (3,577)	68,570 (86,798)	64.3% (4,881)	88,599 (112,150)
Pop implementation	–	0 (0)	–	26,845 (33,980)	–	0 (0)	–	42,959 (54,379)
PPFP implementation	–	0 (0)	–	3,015 (3,816)	–	0 (0)	–	4,825 (6,107)
Pop and PPFP implementation	–	0 (0)	–	29,859 (37,797)	–	0 (0)	–	47,784 (60,486)
Modern contraceptives and interventions implementation TOTAL	–	38,922 (49,269)	–	80,722 (102,180)	–	68,570 (86,798)	–	136,382 (172,636)

Contraception method^a^	2041–2050				2023–2050			
	Without interventions		With Pop and PPFP interventions		Without interventions		With Pop and PPFP interventions	
	Mean % of women using (thousands of users)	Costs in millions of MWK (thousands of USD)	Mean % of women using (thousands of users)	Costs^b^ in millions of MWK (thousands of USD)	Mean % of women using (thousands of users)	Costs in millions of MWK (thousands of USD)	Mean % of women using (thousands of users)	Costs^b^ in millions MWK (thousands of USD)
Pill	2.5% (238)	14,400 (18,228)	3.7% (328)	19,823 (25,093)	2.3% (178)	30,947 (39,174)	3.5% (262)	45,389 (57,454)
IUD	0.5% (52)	9 (11)	2.1% (188)	31 (39)	0.5% (37)	19 (24)	1.6% (129)	72 (92)
Injections	31.8% (3,016)	63,225 (80,031)	34.9% (3,103)	64,830 (82,063)	29.3% (2,281)	137,131 (173,583)	33.1% (2,499)	149,488 (189,225)
Implant	9.9% (940)	6,156 (7,792)	19.6% (1,750)	11,182 (14,155)	8.2% (653)	12,535 (15,867)	15.1% (1,181)	22,828 (28,897)
Male condom	3.5% (334)	6,871 (8,697)	5.6% (498)	10,222 (12,939)	3.3% (258)	15,245 (19,298)	5.5% (412)	24,211 (30,646)
Female sterilization	3.1% (293)	1,368 (1,731)	3.7% (334)	1,454 (1,840)	3.0% (227)	2,957 (3,744)	3.5% (264)	3,395 (4,297)
Other modern	0.3% (29)	622 (787)	0.8% (69)	1,467 (1,857)	0.3% (21)	1,309 (1,657)	0.6% (50)	3,087 (3,907)
Modern contraceptives TOTAL	51.7% (4,900)	92,650 (117,279)	70.4% (6,269)	109,008 (137,985)	46.8% (3,657)	200,143 (253,346)	62.9% (4,796)	248,469 (314,518)
Pop implementation	–	0 (0)	–	50,932 (64,471)	–	0 (0)	–	120,736 (152,830)
PPFP implementation	–	0 (0)	–	5,720 (7,241)	–	0 (0)	–	13,560 (17,164)
Pop and PPFP implementation	–	0 (0)	–	56,652 (71,712)	–	0 (0)	–	134,295 (169,994)
Modern contraceptives and interventions implementation TOTAL	**–**	92,650 (117,279)	–	165,660 (209,697)	–	200,143 (253,346)	–	382,765 (484,513)

^a^ Modern methods only, that is, periodic abstinence, withdrawal and other traditional methods not shown.

^b^ Pop and PPFP interventions starting in 2023, annual costs estimated per population size of 2016 are 1.3 billion MWK and 146 million MWK respectively, which is 2.4 billion MWK and 264 million MKW, respectively, when inflated to 2021 MWK (Appendix 5, Table A5.1); annual costs are then scaled by the total population of women and men aged 15–49 each year

## DISCUSSION

We find that investments in increasing contraception use in Malawi based on the most recent (2016–2020) costed implementation plan from the government of Malawi (Government of Malawi 2015) could have dramatic effects on births and the population size of Malawi to 2050 and beyond. This could reduce current pressures from very rapid population growth on health services, schools, land, and society more broadly (Government of Malawi Ministry of Economic Planning and Development 2012) and help Malawi achieve national goals set out in the growth and development strategy (Government of Malawi 2017) and the sustainable development goals (United Nations Malawi 2023). By rapidly reducing the dependency ratio (number of dependents per adult), estimated to be 44 percent in 2050 with interventions instead of 61 percent as expected without interventions, the investments in contraception outlined in this paper could pay demographic dividends (Cardona et al. 2020). Addressing the remaining unmet need for contraception in Malawi will also deliver women's rights to access contraception to maintain autonomy, enable decisions on when and whether to become pregnant and can reduce health problems incurred by allowing spacing of pregnancies or preventing pregnancies in young women (Guttmacher Institute 2014). Increasing contraception use could also have social and economic benefits via increasing educational attainment (Longwe and Smits 2013; Jiang 2020), and reducing maternal mortality, itself a great benefit (Stover and Ross 2010; Ross and Blanc 2012). Considering the large scale and scope of these benefits the investments in increasing contraception use, at an estimated 0.9 percent of total health expenditure per capita, are modest.

Early mathematical models of contraception have considered contraception failure driving increased numbers of children beyond the desired family size in the USA (Hulka 1969), decision‐making in relation to family planning and population‐related policies (Correa and Beasley 1971), resource allocation for family planning and population programs (Lawrence, Mundigo, and ReVelle 1972), and an economic model of fertility, sex, and contraception (Brunborg 1983). Contemporary international family planning agencies use one of five models to model contraception scale‐up and impact: Adding It Up, Impact 2, ImpactNow, Reality Check, and FamPlan/Lives Save Tool (LiST) (Askew et al. 2017). These are population aggregate models rather than individual‐based simulation models. Our study provides an up‐to‐date projection for Malawi and is the first to be done within a wider framework linking the effects of contraception use, pregnancy, and birth to health and healthcare for all major conditions across the life‐course while also considering resource use and appropriate allocation (The TLOmodel Team 2023). Our individual‐based model of contraception is also a useful addition to other models as it sheds light on parameter values for initiation, switching, and discontinuation of contraceptive methods required to match, and diverge from, UN WPP population projections.

Although our model of contraception in Malawi is much more detailed than other models, it has limitations. We do not model personnel or facility costs to deliver contraception including any additional personnel or facilities that may be required for rapid scale‐up (though we do include implementation costs of the Pop and PPFP interventions, as described in online Appendix 5). We simplify the representation of pregnancy and labor such that for a pregnant woman an event is scheduled in nine months (i.e., for the end of pregnancy) and only then it is decided whether it is or not a live birth or early pregnancy termination. We, therefore, ignore the fact that some women could potentially start (or not) using contraception earlier or be at risk of another pregnancy earlier. Within TLO, it is possible, though far more computationally intensive, to run our analyses together with all other modules including those for pregnancy, labor, and delivery included rather than this simplified representation of pregnancy and births. This is unlikely to change our results much, however, due to the relatively small amount of overall time in the simulation that each woman spends pregnant meaning that including preterm births and early terminations will not alter the overall time a woman is pregnant very much. Our goal was to calibrate our model to the WPP medium variant projection by testing what individual‐level mechanisms are consistent with such a projection (online Appendix 1). For the medium variant, we find one solution and present it. We could repeat this procedure for any projection of course, but there is value in doing it for the medium variant as this is the central (and most likely in the opinion of the WPP) projection. We do not show stochastic uncertainty for our results because, as we explain in our methods section, simulating a large population size (250,000) leads to low variation in results.

We only model interventions as increasing initiation of modern methods, with initiation rates of traditional methods staying the same, and the proportion “not_using” contraception decreasing. An alternative would be to also decrease initiation of traditional methods and also change probabilities of switching from and switching to particular modern methods as a result of the interventions. We also calibrate trajectories of specific methods—with implants the most popular—as per baselines and targets provided in the Malawi CIP (explained in online Appendix 5). This assumption could be altered, which would produce different projections from the ones we present in this paper. It is also possible that efficiency gains could be made across the many CIP demand creation activities of the Pop intervention (Government of Malawi 2015), though alternatively costs could be higher. Empirical evidence from implementation and scale‐up of such activities would be useful to include in further work, once implementation and scale‐up happen in Malawi.

Our model could be further developed via the addition of linear models to predict contraception uptake, discontinuation, and switching based on education, socioeconomic, and demographic characteristics of women (e.g., Dasgupta, Zaba, and Crampin 2015; Nkoka et al. 2020), and potentially also including men. One particularly useful addition could be to incorporate wealth quintile into the model to estimate the effects of reducing unmet contraceptive needs on the poorest. We also plan to incorporate human resource costs and the costs of reusable equipment such as ultrasound into future iterations of our model. Such further work could also include the addition of a model to predict unmet need for contraception and how that could be reduced via more specific interventions for particular groups with high unmet needs. Our ambition is to use the model to estimate the effects of contraception scale‐up on maternal, neonatal, and child health outcomes in Malawi, and on mortality and morbidity more broadly across the life‐course.

To conclude, our calibrated individual‐based model of contraception use and costs in Malawi can produce projections indicative of the kinds of dramatic changes in population that could occur with fully implemented national plans and their continuation in subsequent decades in comparison with the status quo. The modest size of the required investments relative to a range of beneficial outcomes from slower population growth should spur action toward ensuring such investments and successful implementation of contraception scale‐up in Malawi.

Data that support the findings of this study are also available in the Supporting Information of this article.

Our model code is available to view on our website: tlomodel.org (The TLOmodel Team 2023). The code for the contraception model specifically can be found at this link: https://www.tlomodel.org/_modules/tlo/methods/contraception.html#Contraception

## Supporting information

Appendix 1: Calculation and calibration of the age‐specific monthly probability of live birth at the beginning of the simulation
Appendix 2: Explanation of initiation rates calculations
Appendix 3: Switching, Failure, and Discontinuation rates
Appendix 4: Differences by age
Appendix 5: Contraception interventions
Appendix 6: Contraception use and pregnancies – numbers of women

## Data Availability

The data that support the findings of this study were derived from the following resources available in the public domain: Malawi 2010 Demographic and Health Survey (DHS), Malawi 2015–6 DHS https://dhsprogram.com/data/available‐datasets.cfm?ctryid=24
