# Supplementary material for: Modeling Contraception and Pregnancy in Malawi: A Thanzi La Onse Mathematical Modeling Study
Source: Stud Fam Plann. 2023 Dec 21;54(4):585–607. doi: 10.1111/sifp.12255 (PMC10941698; doi:10.1111/sifp.12255)
Supplement: Supplementary file 1 — Appendix 1: Calculation and calibration of the age‐specific monthly probability of live birth at the beginning of the simulation Appendix 2: Explanation of initiation rates calculations Appendix 3: Switching, Failure, and Discontinuation rates Appendix 4: Differences by age Appendix 5: Contraception interventions Appendix 6: Contraception use and pregnancies – numbers of women [file SIFP-54-585-s001.docx]

# Appendix 1: Calculation and calibration of age-specific monthly probability of live birth at the beginning of the simulation.

This analysis uses the individual recode dataset MWIR61FL.DTA from the Malawi DHS 2010 survey, downloaded from the DHS website with permission (<https://dhsprogram.com/data/available-datasets.cfm> accessed 15th October 2019) and was done by Tim Colbourn in Stata using Fertility_v4.do.

The objective of this analysis and calibration is to determine the age-specific monthly risks of pregnancy at the beginning of the simulation to ensure that the probability of a woman having a live birth matches the United Nations World Population Prospects (WPP) age-specific fertility rate value for the same year.

We start with the average pregnancies per year in those not using contraception by year of age for women aged 15-49 in 2010, shown in the second column of Table A1.2 (Pregnancy_NotUsing_In_2010 in the ResourceFile_Contraception.xlsx). This was calculated from the DHS survey data using the data on births in the last year for each woman, and estimates of the relative risk of pregnancy given each contraceptive method and the proportion of women using each contraceptive method, using the following formula:

[1] $F_{a}=F_{0a}r_{1}p_{1a}+F_{0a}r_{2}p_{2a}+\ldots+F_{0a}r_{k}p_{ka}$

Where $F_{a}$ is total fertility of a woman aged *a,* $F_{0a}$ is baseline fertility at age *a* (the parameter we are interested in), $r_{1}$,$r_{2}{\ldots r}_{k}$ are the risks of pregnancy in one year of use for contraceptive methods 1, 2…*k* , i.e. 1 minus the effectiveness of the contraceptive method (see Table A1.1 below), and $p_{1a}$, $p_{2a}$ …. $p_{ka}$ are the proportion of women at age *a* using contraceptive methods 1, 2, …, *k*  (data obtained from Malawi DHS 2010 survey, Table A1.2).

## Table A1.1 Effectiveness of contraceptive methods

| Contraceptive method | Effectiveness (one year of use) | *r* (risk of pregnancy in one year of use† = 1 - effectiveness). |
| --- | --- | --- |
| pill | 0.9769 | 0.0231 |
| IUD | 1 | 0 |
| injection (DMPA) | 0.9936 | 0.0064 |
| implant | 0.9989 | 0.0011 |
| male condom | 0.986 | 0.0140 |
| female sterilization | 1 | 0 |
| other modern (e.g. female condom) | 1 | 0 |
| periodic abstinence | 0.9934 | 0.0066 |
| withdrawal | 0.9571 | 0.0429 |
| other traditional | 0.9409 | 0.0591 |

† These are the failure rates derived from the DHS contraceptive calendar data i.e. the failure parameter detailed in Appendix 3, Table A3.3

$F_{0a}$, baseline fertility at age *a* (i.e. fertility without contraception use) is calculated by rearranging equation [1] to:

[2] $F_{0a}={{F'}_{a}}/{\sum_{i=1}^{k} {r_{i}p}_{ia}}$

Where ${F'}_{a}$ is the observed total fertility (births in the last year, according to Malawi DHS 2010 data) per woman aged *a*, and $\sum_{i=1}^{k} {r_{i}p}_{ia}$ is the sum risk of pregnancy expected at age *a* weighted by the proportion using each contraception method (1 to *k*) across all women aged *a*. We multiply the ‘birth in the last year’ variable by 1.25 to estimate pregnancies in the last year given 20% are likely to end in miscarriages, abortions and stillbirths which are likely to not be counted as births (1 is 80% of 1.25).

Following this, the calibration to the expected live births per year by age for each women to match the WPP data is done in two stages:

1. The scaling_factor_on_monthly_risk_of_pregnancy parameter is used to induce the correct number of age-specific births initially, given the initial pattern of contraceptive use by age set at the beginning of the simulation, using DHS data, as shown in Table A1.2. By 5-year age group these scaling factors are: [1.227, 0.7191, 0.6632, 0.84945, 0.86135, 0.645, 0.941] i.e. for 15-19 year old women we multiply the average number of pregnancies per year in those not using contraception (column 2 of Table A1.2) by 1.227, for 20-24 year old women we multiply by 0.7191 , …, and for 45-49 year old women we multiply by 0.941. These scaling factors were found via trial and subsequent error and visual inspection of model live births against WPP data.

2. Trends over time in the risk of starting (time_age_trend_in_initiation) and stopping (time_age_trend_in_stopping) contraception are used to induce the correct trend in the number of births expected according to the medium variant WPP population projection. This involves multiplying the contraception initiation rates and stopping rates by a scaling factor for each calendar year from 2011 (the year after the start of the simulation) to 2100 (latest possible year and end of WPP projection). For initiation, the scaling factors are calculated using the formula [3]:

[3] $e^{0.05\left( min\left( 2020-2010,year-2010 \right)*max\left( 1,e^{0.01\left( year-2020 \right)} \right) \right)}$

Where *e* is the base of the natural logarithm (2.7182818), 2020 and 2010 are calendar years, *year* is the calendar year in question that we want the scaling factor for, and *min* and *max* refer to the minimum or maximum of the two numbers separated by commas in the brackets after them.

This formula gives a different result for years before 2020 and years after 2020 (an inflection point), as shown in Figure A1.1. This was required to calibrate to the curving WPP population projection for Malawi.

For stopping, the scaling factors are calculated using the formula [4], with the same notation as formula [3] above, and are shown in Figure A1.2.

[4] $e^{-0.05\left( min\left( 2020-2010,year-2010 \right)*max\left( 1,e^{-0.01\left( year-2020 \right)} \right) \right)}$

Trends over time in the risk of starting and stopping contraception are respectively further modified by 5 year age groups to calibrate to expected trends in live births over time, via the parameters _init_over_time_modification_by_age 1.0 / [1.0, 0.6, 0.6, 0.6, 0.6, 0.6, 0.6] = [1, 1.6667, 1.6667, 1.6667, 1.6667, 1.6667, 1.6667] and _discont_over_time_modification_by_age [1.0, 0.5, 0.5, 0.5, 0.5, 0.5, 0.5] for ages 15-19, 20-24, 25-29, 30-34, 35-39, 40-44, 45-49 respectively, i.e. initiation rate is decreased by 40% for women aged 15-19 relative to women in older age groups and the discontinuation rate halved for women aged 20-49 relative to women aged 15-19.

In the paper (Figure 2) we calibrate to WPP 2019 births and population. In Figure A1.3 below we show how our model calibration compares to WPP 2022 births and population, and show that our model produces birth and population estimates that are still within the bounds of the uncertainty interval of the WPP 2022 projections. This is not surprising given the WPP 2022 estimates for Malawi are not so different to the WPP 2019 estimates out to 2030 (medium variant projection: WPP 2019: 24,849,443 people, WPP 2022: 24,644,069 people; WPP 2022 is 0.2 million less, which is <1% less) and 2050 (medium variant projection: WPP 2019: 38,142,971 people, WPP 2022: 36,859,454 people; WPP 2022 is 1.3 million less, which is ~3% less). We do not use WPP 2022 in the main paper because the main Thanzi La Onse demography module and calibrations use the WPP 2019 data.

## Table A1.2: Proportions of women not using contraception and using each contraception method by age in years from DHS data (Pregnancy_NotUsing_In_2010 and Method_Use_In_2010 in ResourceFile_Contraception.xlsx) – grey cells add to 100% for each row (age)

| Age | Average pregnancies per year in those not using contraception | not using contraception | pill | IUD | injection | implant | male condom | female sterilization | other modern | periodic abstinence | withdrawal | other traditional |
| --- | --- | --- | --- | --- | --- | --- | --- | --- | --- | --- | --- | --- |
| 15 | 0.015 | 98.2% | 0.0% | 0.0% | 0.5% | 0.0% | 1.0% | 0.0% | 0.0% | 0.1% | 0.2% | 0.0% |
| 16 | 0.078 | 95.6% | 0.0% | 0.1% | 1.9% | 0.0% | 2.0% | 0.0% | 0.0% | 0.3% | 0.0% | 0.1% |
| 17 | 0.148 | 91.7% | 0.1% | 0.0% | 4.1% | 0.0% | 2.8% | 0.0% | 0.0% | 0.3% | 0.7% | 0.1% |
| 18 | 0.298 | 84.4% | 0.7% | 0.0% | 10.3% | 0.0% | 4.0% | 0.0% | 0.0% | 0.0% | 0.6% | 0.0% |
| 19 | 0.402 | 73.9% | 1.9% | 0.0% | 18.2% | 0.0% | 3.8% | 0.0% | 0.0% | 0.1% | 1.4% | 0.3% |
| 20 | 0.533 | 69.3% | 1.1% | 0.0% | 21.8% | 0.0% | 4.1% | 0.4% | 0.0% | 0.2% | 2.2% | 0.3% |
| 21 | 0.552 | 64.4% | 1.7% | 0.1% | 27.3% | 0.0% | 3.7% | 0.1% | 0.0% | 0.1% | 0.7% | 0.6% |
| 22 | 0.586 | 63.5% | 1.9% | 0.0% | 26.4% | 0.0% | 3.6% | 0.4% | 0.0% | 0.6% | 1.6% | 0.7% |
| 23 | 0.569 | 64.3% | 0.7% | 0.2% | 26.5% | 0.0% | 3.9% | 0.3% | 0.0% | 0.8% | 1.2% | 0.7% |
| 24 | 0.599 | 58.8% | 2.1% | 0.1% | 28.6% | 0.0% | 3.0% | 1.3% | 0.0% | 0.8% | 2.3% | 0.9% |
| 25 | 0.623 | 56.5% | 2.4% | 0.2% | 30.6% | 0.0% | 3.8% | 1.3% | 0.0% | 0.7% | 2.0% | 0.6% |
| 26 | 0.570 | 55.2% | 2.7% | 0.1% | 32.4% | 0.0% | 3.6% | 2.0% | 0.0% | 0.3% | 1.4% | 0.2% |
| 27 | 0.605 | 54.3% | 3.1% | 0.1% | 31.6% | 0.0% | 3.3% | 1.5% | 0.0% | 0.5% | 1.9% | 0.5% |
| 28 | 0.550 | 55.6% | 4.0% | 0.3% | 29.4% | 0.0% | 2.3% | 3.0% | 0.0% | 0.8% | 1.7% | 0.2% |
| 29 | 0.556 | 54.6% | 3.2% | 0.1% | 28.0% | 0.0% | 3.0% | 4.8% | 0.1% | 0.7% | 1.4% | 1.0% |
| 30 | 0.474 | 53.6% | 2.3% | 0.0% | 27.5% | 0.0% | 3.2% | 5.8% | 0.1% | 0.4% | 2.5% | 2.0% |
| 31 | 0.475 | 55.7% | 4.0% | 0.3% | 25.0% | 0.0% | 2.6% | 6.6% | 0.0% | 0.6% | 1.9% | 0.3% |
| 32 | 0.513 | 53.4% | 2.6% | 0.3% | 26.3% | 0.0% | 2.8% | 8.9% | 0.0% | 1.0% | 1.6% | 0.7% |
| 33 | 0.514 | 52.5% | 3.5% | 0.2% | 23.7% | 0.0% | 1.9% | 12.1% | 0.0% | 0.7% | 2.1% | 1.2% |
| 34 | 0.461 | 53.6% | 2.8% | 0.8% | 20.1% | 0.0% | 3.3% | 12.8% | 0.2% | 0.8% | 2.2% | 1.5% |
| 35 | 0.440 | 51.5% | 2.9% | 0.2% | 23.5% | 0.0% | 2.5% | 13.4% | 0.2% | 0.6% | 2.1% | 1.6% |
| 36 | 0.420 | 49.7% | 3.4% | 0.0% | 20.1% | 0.0% | 2.5% | 16.9% | 0.2% | 1.1% | 2.9% | 1.3% |
| 37 | 0.400 | 55.1% | 1.4% | 0.4% | 18.0% | 0.0% | 1.8% | 15.8% | 0.2% | 0.6% | 3.8% | 1.2% |
| 38 | 0.368 | 54.2% | 3.2% | 0.2% | 15.1% | 0.0% | 2.0% | 19.3% | 0.0% | 0.4% | 2.0% | 2.2% |
| 39 | 0.376 | 52.4% | 3.3% | 0.8% | 12.5% | 0.0% | 3.1% | 22.0% | 0.8% | 1.0% | 2.3% | 0.8% |
| 40 | 0.340 | 50.2% | 2.0% | 0.0% | 15.0% | 0.0% | 2.4% | 23.9% | 0.0% | 1.2% | 1.4% | 3.2% |
| 41 | 0.179 | 56.0% | 2.4% | 0.0% | 9.8% | 0.0% | 1.5% | 25.6% | 0.0% | 0.9% | 1.2% | 2.1% |
| 42 | 0.197 | 58.1% | 1.3% | 0.0% | 10.5% | 0.0% | 2.0% | 23.0% | 0.0% | 1.0% | 1.8% | 1.8% |
| 43 | 0.174 | 56.6% | 1.1% | 0.0% | 13.2% | 0.0% | 1.9% | 22.6% | 0.0% | 2.3% | 0.8% | 0.8% |
| 44 | 0.147 | 58.1% | 0.7% | 0.0% | 7.2% | 0.0% | 0.7% | 28.5% | 0.0% | 0.7% | 0.7% | 3.1% |
| 45 | 0.075 | 60.2% | 0.6% | 0.0% | 5.5% | 0.0% | 3.3% | 24.6% | 0.0% | 1.2% | 2.4% | 1.8% |
| 46 | 0.079 | 55.9% | 0.5% | 0.3% | 6.6% | 0.0% | 0.5% | 29.6% | 0.0% | 1.1% | 1.1% | 3.8% |
| 47 | 0.039 | 64.3% | 0.6% | 0.0% | 6.0% | 0.0% | 1.1% | 25.4% | 0.0% | 0.0% | 1.4% | 1.1% |
| 48 | 0.051 | 65.1% | 0.0% | 0.3% | 3.8% | 0.0% | 0.3% | 26.4% | 0.0% | 0.6% | 1.2% | 2.3% |
| 49 | 0.036 | 70.6% | 0.8% | 0.0% | 3.2% | 0.0% | 0.4% | 21.8% | 0.0% | 0.0% | 1.2% | 2.0% |

## Figure A1.1 Scaling factor for contraception initiation over time to match WPP medium variant population projection.

## Figure A1.2 Scaling factor for contraception discontinuation over time to match WPP medium variant population projection.


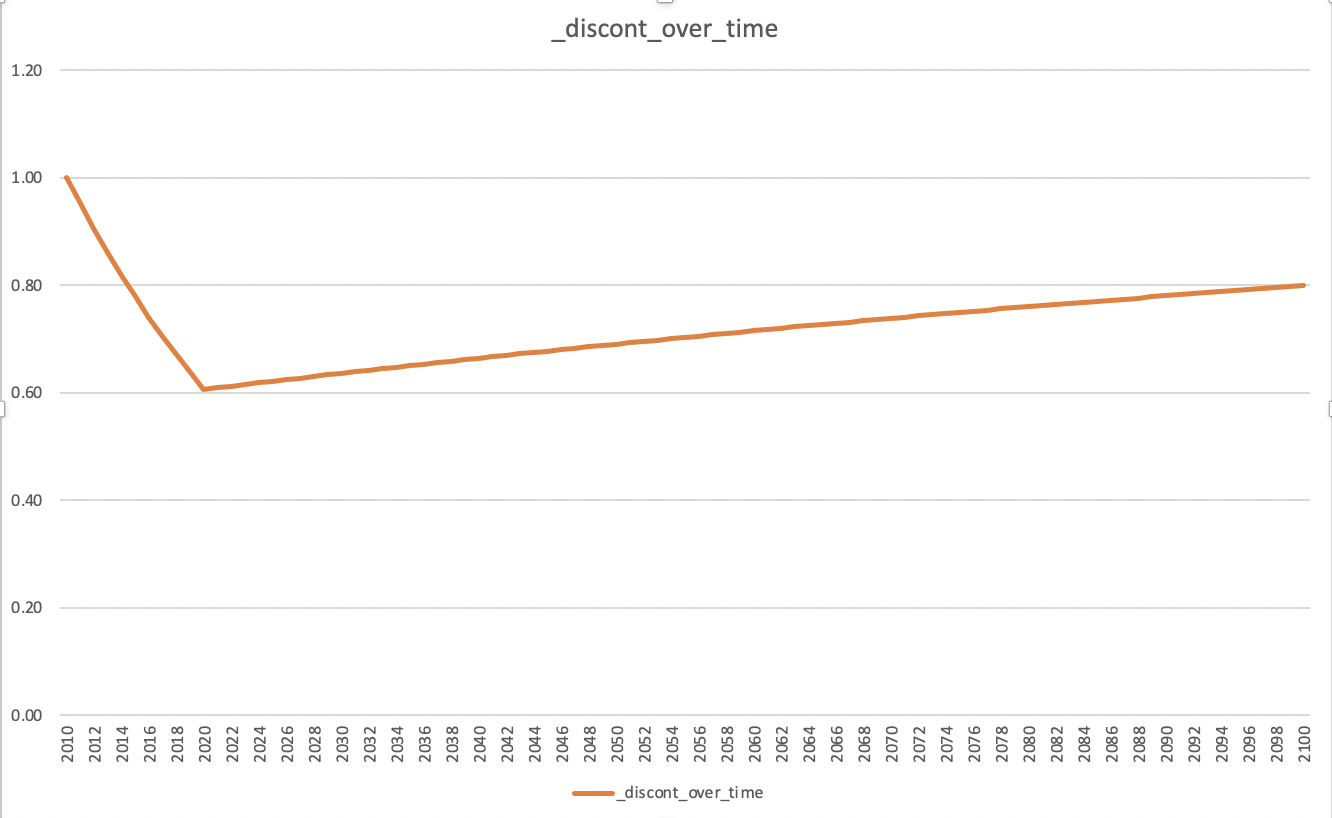


**Figure A1.3 Model births and population results compared to World Population Prospects (WPP) 2022**

**(A)** Number of births, **(B)** Population pyramid in 2018, **(C)** Population pyramid in 2050,

**(D)** Population size;

Calibration (without Pop and PPFP interventions)


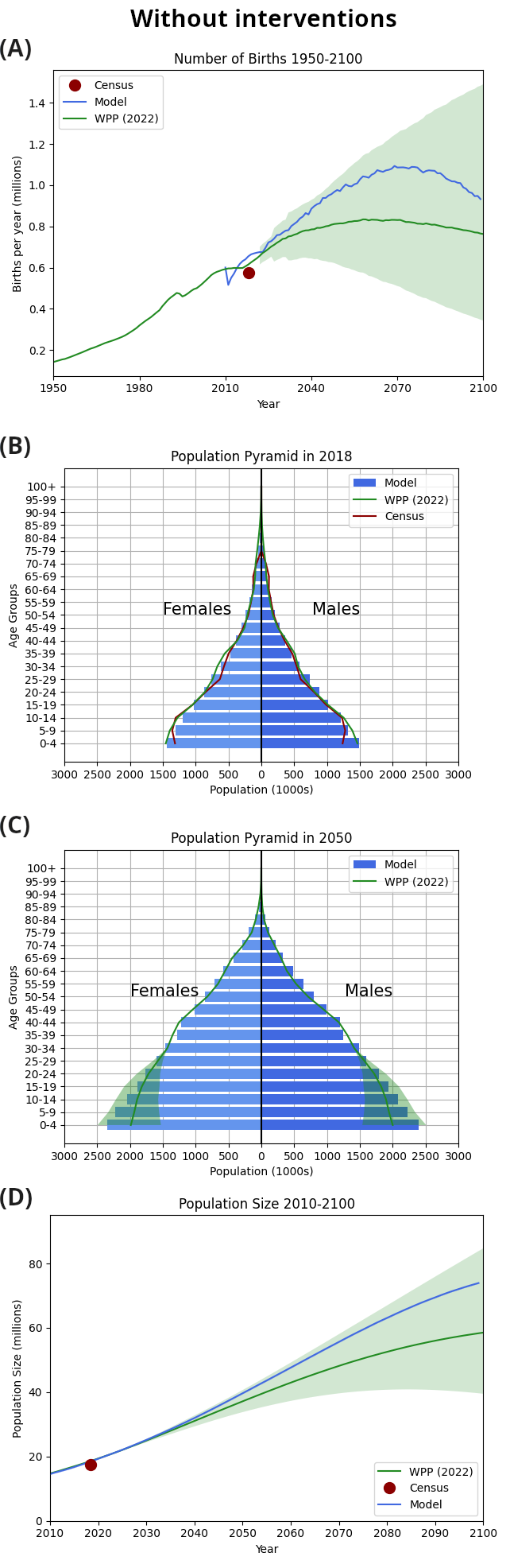


# Appendix 2: Explanation of initiation rates calculations

This analysis uses the eventsfile2010.dta dataset from the contraceptive calendar data from the Malawi DHS 2010. The analysis follows the guidance from the DHS program, contained in the document: ‘DHS Contraceptive Calendar Tutorial, version 2 September 2018’.

Initiation_ByMethod

Monthly initiation rates from not using contraception, *I_m_*, for each contraception method, *m*, (Table A2.1) were calculated using the following formula:

[3] $I_{m}=\frac{\sum_{j=1}^{N} {i_{m}}^{\left( j \right)}}{\sum_{j=1}^{N} t^{\left( j \right)}}$

where *m* indicates one of *k* contraception methods, $i_{m}^{\left( j \right)}$ is an indicator variable of a contraception initiation event for the method *m* denoted as the transition of the woman *j* from not using contraception the previous month (marked 0 on the DHS contraceptive calendar) to using the particular contraception method the following month; *t^(j)^* is the time at ‘risk’ (in months) of the contraception initiation i.e. the number of months of contraceptive calendar data for woman *j* during which she was not using contraception (pregnancies, births and terminations are excluded from this denominator). The contraception initiation events and time at risk are summed for all women (*N*) in the DHS contraception calendar datasets.

Initiation_AfterBirth

Monthly initiation rates following end of pregnancy (birth, or termination due to miscarriage, abortion, or stillbirth), *I'_m_,* for each contraception method, *m*, were calculated using the following formula:

[4] ${I'}_{m}=\frac{\sum_{j=1}^{N} {i'}_{m}^{\left( j \right)}}{\sum_{j=1}^{N} {t'}^{\left( j \right)}}$

where *m* indicates one of *k* contraception methods, ${i'}_{m}^{\left( j \right)}$ is an indicator variable of a contraception initiation event for the method *m* denoted as the transition of the women *j* from pregnancy, birth or termination the previous month (marked P, B, or T on the DHS contraceptive calendar) to using the particular contraception method the following month; *t'^(j)^* is the single month at ‘risk’ of contraception initiation for woman *j* immediately after birth or termination of pregnancy and no month at ‘risk’ otherwise. The contraception initiation events and month at risk are summed for all women (*N*) in the DHS contraception calendar datasets. As there is a single month at ‘risk’ of contraception initiation after birth for each woman, *I'_m_* (Initiation_AfterBirth) is a monthly initiation rate (Table A2.2). Please note these rates are low as they are just for the month after pregnancy and then the remainder (1 - Initiation_AfterBirth = 99.7%, Table A2.2) who ‘initiate’ to ‘not_using’ i.e. 1 - $\sum_{m=1}^{k} {I'}_{m}$ are then subject to the usual monthly initiation rates *I_m_* as per the model of contraception and pregnancy shown in Figure 1.

## Changes over time, and by age

Initiation rates are adjusted over time as described in Appendix 1. Initiation rates are also adjusted by women’s age as described in Appendix 4.

## Table A2.1: Monthly Initiation rates to each type of contraception from not using (remainder is not using), 2005-2010 DHS data (Initiation_ByMethod in ResourceFile_Contraception.xlsx)

|  | pill | IUD | injection | implant | male condom | female sterilization | other modern | periodic abstinence | withdrawal | other traditional | not using |
| --- | --- | --- | --- | --- | --- | --- | --- | --- | --- | --- | --- |
| monthly | 0.040% | 0.002% | 0.410% | 0.012% | 0.079% | 0.027% | 0.003% | 0.009% | 0.036% | 0.016% | 99.393% |

## Table A2.2: Initiation rates to each type of contraception in the month after pregnancy, birth or termination (remainder is not using), 2005-2010 DHS data (Initiation_AfterBirth in ResourceFile_Contraception.xlsx)

|  | pill | IUD | injection | implant | male condom | female sterilization | other modern | periodic abstinence | withdrawal | other traditional | not using |
| --- | --- | --- | --- | --- | --- | --- | --- | --- | --- | --- | --- |
| monthly | 0.022% | 0.001% | 0.180% | 0.005% | 0.021% | 0.072% | 0.001% | 0.011% | 0.018% | 0.015% | 99.726% |

# Appendix 3: Switching, Failure, and Discontinuation rates

This analysis uses the eventsfile.dta dataset from the contraceptive calendar data from the Malawi DHS 2016. The analysis (reproducible in the Stata file ‘failure discontinuation switching rates.do’) follows the guidance from the DHS program, contained in the document: ‘DHS Contraceptive Calendar Tutorial, version 2 September 2018’. Contraception switching, discontinuation and failure rates were calculated as competing risks i.e. when a woman is on contraception she could stop using it for many reasons including switching to another method, discontinuing because of wanting to get pregnant, or due to side effects (for example), or due to the method failing and her becoming pregnant. The risks of each of these happening compete with each other so that the incidence rate of each takes account of these alternative possibilities. We used the **stcompet** command in Stata.

Prob_Switch_From

Monthly switching rates, *S_m_*, from each contraception method, *m*, were calculated using the **stcompet** command in Stata, which uses the following formula^1,2^ (please see Prob_Switch_From_And_To below for the method the woman switches to):

[5] $S_{m}\left( t \right)=\sum_{j:t_{\left( j \right)}\leq t} M_{m}\left( t_{\left( j-1 \right)} \right)\frac{c_{rj}}{N_{j}}$

where *j* denotes an individual woman on contraception in the dataset, *t* is the time (month) when switching occurs, $j:t_{\left( j \right)}\leq t$ is the time at ‘risk’ (in months) of the contraception switching i.e. the number of months of contraceptive calendar data for woman *j* during which she was using the contraception method she switched from; $M_{m}\left( t_{\left( j-1 \right)} \right)$ is the Kaplan-Meier estimate of the overall survival function of staying on the same contraception method *m* (see formula [6]); this is scaled by $\frac{c_{1j}}{N_{j}}$ which is the proportion of the competing risk of switching contraceptive methods due to switching to another contraception method for woman *j* (i.e. competing risk $c_{rj}$*, r=1*)*,* among all competing risks for woman *j* (i.e. cumulative risk of switching contraceptive method *m* due to any competing risk *r,* $N_{j}=\sum_{r=1}^{n} c_{rj}$)_._ In this model *r*=1 is switching to another contraception method and *r*=2 is any other change in contraception status (i.e. *n*=2, *r* can either be 1 or 2).

Monthly contraception switching rates were calculated for each contraceptive method using the formula [5] and are used in the model (Table A3.1). Quarterly switching rates could be obtained by multiplying these by 4 and annual switching rates could be obtained by multiplying these by 12. We are not using the option of calculating standard errors surrounding the cumulative incidence of the competing risks model (for switching, or failure or discontinuation below), though this is an option in the **stcompet** Stata command.^1,2^ This could be used in future stochastic versions of the contraception model.

Kaplan-Meier estimate^2^ of the overall survival function of staying on contraception method *m* during time $0\leq t\leq t_{m}$:

[6] $M_{m}\left( t_{\left( j-1 \right)} \right)=\prod_{j:t_{\left( j \right)}\leq t} \left( 1-\frac{c_{rj}}{N_{j}} \right),0\leq t\leq t_{t}$

where $t_{t}$ is the total time (months) of the survival analysis period.

Prob_Switch_From_And_To

The DHS 2016 contraceptive calendar data were used to determine the contraception method a women switched to after switching from a particular method. This switching matrix (Table A3.2) was calculated directly from the data as the proportion of the total switches from each of the 9 contraception methods (rows of Table A3.2) to each of the 10 methods (columns of Table A3.2). Female sterilization is permanent, hence it is not possible to switch from this method to another.

Failure_ByMethod

Monthly failure rates, *F_m_*, for each contraception method, *m*, were calculated similar to contraception switching rates above, using the **stcompet** command in Stata. Formula [5] can be substituted as:

[7] $F_{m}\left( t \right)=\sum_{j:t_{\left( j \right)}\leq t} M_{m}\left( t_{\left( j-1 \right)} \right)\frac{c_{rj}}{N_{j}}$

where everything as in formula [5] and associated text except *r*=1 is failure of the contraception method and competing risks *r*=2 to *r*=7 are discontinuation of contraception due to a number of different reasons which sum together as the discontinuation rate (see Discontinuation_ByMethod below) (i.e. *n*=7, *r* can either be 1, 2, 3, 4, 5, 6 or 7).

Monthly contraception failure rates were calculated for each contraceptive method using the formula [7] and are used in the model (Table A3.3). These monthly rates, multiplied by 12 are consistent with the expected annual rates of failure of the contraceptive methods shown in the right hand column of Table A1.1 in Appendix 1.

Discontinuation_ByMethod

Contraception discontinuation is determined from the same model as contraception failure above. In formula [8] below, the Kaplan-Meier estimate is scaled by the proportion of the sum of competing risks of discontinuation for all reasons for discontinuation (2 = “desire to become pregnant”, 3 = “other method related reason”, 4 = “side effects”, 5 = “wanted more effective method”, 6 = “other fertility related reasons”, 7 = “other reason / don’t know”) among all competing risks; *r* could also = 1 (failure of the contraception method, as above) as a competing risk. Monthly discontinuation rate, D*_m_*, for each contraception method, *m*, are:

[8] $D_{m}\left( t \right)=\sum_{j:t_{\left( j \right)}\leq t} M_{m}\left( t_{\left( j-1 \right)} \right)\frac{\sum_{r=2}^{r=7} c_{rj}}{N_{j}}$

Monthly contraception discontinuation rates were calculated for each contraceptive method using the formula [8] and are used in the model (Table A3.4).

## Table A3.1 Monthly Switching rates from each contraceptive method to a new method (Prob_Switch_From in ResourceFile_Contraception.xlsx)

|  | pill | IUD | injection | implant | male condom | female sterilization | other modern | periodic abstinence | withdrawal | other traditional |
| --- | --- | --- | --- | --- | --- | --- | --- | --- | --- | --- |
| monthly | 1.09% | 0.15% | 0.30% | 0.09% | 1.14% | 0.00% | 0.27% | 0.75% | 1.38% | 2.72% |

## Table A3.2 Switching matrix: proportion of those who switch from each contraception method that switch to each new method (Prob_Switch_From_And_To in ResourceFile_Contraception.xlsx)

|  |  | **switch to** | | | | | | | | | |
| --- | --- | --- | --- | --- | --- | --- | --- | --- | --- | --- | --- |
|  |  | pill | IUD | injection | implant | male condom | female sterilization | other modern | periodic abstinence | withdrawal | other traditional |
| **switch from** | pill | 0.00% | 0.00% | 54.03% | 21.77% | 10.48% | 5.65% | 2.42% | 0.81% | 4.03% | 0.81% |
|  | IUD | 12.50% | 0.00% | 37.50% | 25.00% | 12.50% | 12.50% | 0.00% | 0.00% | 0.00% | 0.00% |
|  | injection | 26.28% | 2.67% | 0.00% | 44.77% | 10.69% | 7.57% | 0.89% | 0.89% | 4.45% | 1.78% |
|  | implant | 20.59% | 2.94% | 63.24% | 0.00% | 10.29% | 2.94% | 0.00% | 0.00% | 0.00% | 0.00% |
|  | male condom | 8.29% | 0.49% | 59.51% | 22.44% | 0.00% | 2.93% | 0.49% | 0.98% | 4.88% | 0.00% |
|  | other modern | 0.00% | 0.00% | 100.00% | 0.00% | 0.00% | 0.00% | 0.00% | 0.00% | 0.00% | 0.00% |
|  | periodic abstinence | 0.00% | 0.00% | 33.33% | 66.67% | 0.00% | 0.00% | 0.00% | 0.00% | 0.00% | 0.00% |
|  | withdrawal | 3.70% | 1.85% | 51.85% | 29.63% | 11.11% | 1.85% | 0.00% | 0.00% | 0.00% | 0.00% |
|  | other traditional | 0.00% | 0.00% | 73.91% | 14.49% | 5.80% | 5.80% | 0.00% | 0.00% | 0.00% | 0.00% |

## Table A3.3 Monthly Failure rates from each contraceptive method to Pregnancy (Failure_ByMethod in ResourceFile_Contraception.xlsx)

|  | pill | IUD | injection | implant | male condom | female sterilization | other modern | periodic abstinence | withdrawal | other traditional |
| --- | --- | --- | --- | --- | --- | --- | --- | --- | --- | --- |
| monthly | 0.19% | 0.00% | 0.05% | 0.01% | 0.12% | 0.00% | 0.00% | 0.06% | 0.36% | 0.49% |

## Table A3.4 Monthly Discontinuation rates from each contraceptive method to not using (Discontinuation_ByMethod in ResourceFile_Contraception.xlsx)

|  | pill | IUD | injection | implant | male condom | female sterilization | other modern | periodic abstinence | withdrawal | other traditional |
| --- | --- | --- | --- | --- | --- | --- | --- | --- | --- | --- |
| monthly | 4.96% | 1.11% | 3.34% | 0.63% | 5.04% | 0.00% | 3.33% | 4.23% | 4.23% | 4.98% |
|  |  |  |  |  |  |  |  |  |  |  |

# Appendix 4: Differences by age

## Relative Risk (RR) of contraceptive failure

rr_fail_under25 **= 2.2** for women younger than 25

This was calculated using the results of an analysis of 10 East African DHS contraceptive calendar surveys by the Guttmacher institute (Table A4.1, see Table 9, page 52 of the report^3^). Women under 25 had statistically significantly higher failure rates than those over 25 for the following methods of contraception: pill, injection, and periodic abstinence, and higher failure rates for almost all of the other contraception methods with data: implant, male condom and withdrawal (see first rows of Table A4.1 and the row below the table). The relative risk of failure when under 25 was then calculated as the average of the relative risks of failure in women under 25 compared to women over 25 for each method (except IUD and implant for which there were not enough data) weighted by the proportion of women using this method among all women aged 15-49 years in Malawi according to the 2010 DHS data (row below Table A4.1).

## Proportional incremental change in Initiation_ByMethod rate for each age in years of the woman:

Initiation_ByAge

Following advice in the DHS Contraception Calendar data tutorial^1^, Cox regression of contraception initiation (_d in survival time stset data in Stata) with age as a covariate was undertaken to estimate how the initiation rate (see Appendix 2, formula [3]) varies by age. Given initiation rates vary non-linearly with increasing age we first used age and age squared (which was significantly associated with initiation) and then used an automated tool in Stata (the fracpoly command) to estimate the two best fitting powers of age (exact, not rounded to the nearest year). The results of these models are plotted in Figure A4.1, and shown in Table A4.2 as the parameter r_init1_age: the proportional incremental change in Initiation_ByMethod rate for each age in years of the woman relative to the average Initiation_ByMethod rate for all women.

## Proportional incremental change in Discontinuation_ByMethod rate for each age in years of the woman:

Discontinuation_ByAge

Linear regression of contraception discontinuation rate with age as a covariate was undertaken to estimate how the discontinuation rate (see Appendix 3, formula [8]) varies by age. Given discontinuation rates vary non-linearly with increasing age we first used age and age squared (which was significantly associated with discontinuation) and then used an automated tool in Stata (the fracpoly command) to estimate the two best fitting powers of age (exact, not rounded to the nearest year). The results of these models are plotted in Figure A4.2, and shown in Table A4.3 as the parameter r_discont_age: the proportional incremental change in Discontinuation_ByMethod rate for each age in years of the woman relative to the average Discontinuation_ByMethod rate for all women.

## Table A4.1 Twelve-month failure rates (number of failures per 100 episodes of use) by method, according to demographic and socioeconomic characteristics, for Eastern Africa^3^

| Demographic and socioeconomic factors* | | **pill** | | | **IUD** | | | **injection** | | | **implant** | | | **male condom** | | | **periodic abstinence** | | | **withdrawal** | | |
| --- | --- | --- | --- | --- | --- | --- | --- | --- | --- | --- | --- | --- | --- | --- | --- | --- | --- | --- | --- | --- | --- | --- |
|  |  | **Failure rate** | 95% CI (–/+) | | **Failure rate** | 95% CI (–/+) | | **Failure rate** | 95% CI (–/+) | | **Failure rate** | 95% CI (–/+) | | **Failure rate** | 95% CI (–/+) | | **Failure rate** | 95% CI (–/+) | | **Failure rate** | 95% CI (–/+) | |
| **Age** | |  |  |  |  |  |  |  |  |  |  |  |  |  |  |  |  |  |  |  |  |  |
|  | <25 | **6.8** | 5.5 | 8.1 | **-** | - | - | **3.1** | 2.3 | 3.9 | **(1.5)** | 0 | 3.8 | **4.2** | 2.8 | 5.6 | **18.7** | 14.6 | 22.8 | **17.8** | 14 | 21.6 |
|  | ≥25 | **3.7** | 3 | 4.4 | **0.5** | 0 | 1.1 | **1.3** | 1 | 1.7 | **0.4** | 0 | 0.8 | **2.8** | 1.7 | 4 | **10.3** | 8.1 | 12.5 | **13** | 10.3 | 15.7 |
| **Marital status** | |  |  |  |  |  |  |  |  |  |  |  |  |  |  |  |  |  |  |  |  |  |
|  | Never married | **9.1** | 4.3 | 14 | **-** | - | - | **2.4** | 0.7 | 4.1 | **-** | - | - | **2.1** | 1.1 | 3 | **13.6** | 7.6 | 19.7 | **-** | - | - |
|  | ever married | **4.5** | 3.9 | 5.1 | **1.3** | 0 | 2.9 | **1.8** | 1.5 | 2.2 | **0.6** | 0 | 1.1 | **4.4** | 3.1 | 5.7 | **12.9** | 10.6 | 15.2 | **14.6** | 12.2 | 16.9 |
| **Parity** | |  |  |  |  |  |  |  |  |  |  |  |  |  |  |  |  |  |  |  |  |  |
|  | 0–2 children | **5.3** | 4.4 | 6.1 | **(2.7)** | 0 | 6.4 | **2.6** | 2 | 3.2 | **1.1** | 0 | 2.5 | **3.4** | 2.4 | 4.4 | **15.1** | 11.9 | 18.3 | **14.8** | 11.4 | 18.2 |
|  | ≥3 children | **4.1** | 3.2 | 5 | **(0.1)** | 0 | 0.2 | **1.4** | 1 | 1.8 | **0.3** | 0 | 0.7 | **3.8** | 1.9 | 5.7 | **10.8** | 8.2 | 13.4 | **14.3** | 11.3 | 17.4 |
| **Contraceptive intention** | |  |  |  |  |  |  |  |  |  |  |  |  |  |  |  |  |  |  |  |  |  |
|  | For spacing | **4.7** | 4 | 5.5 | **1.4** | 0 | 4.1 | **2.2** | 1.7 | 2.7 | **1** | 0 | 2.1 | **3.5** | 2.5 | 4.4 | **13.8** | 11 | 16.5 | **14.7** | 11.9 | 17.4 |
|  | For limiting | **4.8** | 3.7 | 5.9 | **(0.1)** | 0 | 2.4 | **1.4** | 0.9 | 1.8 | **0.1** | 0 | 0.3 | **3.8** | 1.5 | 6.1 | **11.6** | 8.1 | 15.1 | **14.3** | 10.4 | 18.2 |
| **Wealth** | |  |  |  |  |  |  |  |  |  |  |  |  |  |  |  |  |  |  |  |  |  |
|  | Lower three quintiles | **4.6** | 3.7 | 5.4 | **-** | - | - | **2.2** | 1.7 | 2.7 | **0.1** | 0 | 0.4 | **6** | 3.8 | 8.2 | **17.5** | 13.9 | 21.1 | **15.8** | 12.7 | 18.8 |
|  | Upper two quintiles | **4.9** | 4 | 5.8 | **0.6** | 0 | 1.4 | **1.6** | 1.1 | 2 | **0.9** | 0 | 1.8 | **2.4** | 1.6 | 3.2 | **9** | 6.6 | 11.5 | **13.2** | 10.2 | 16.2 |
| **Residence** | |  |  |  |  |  |  |  |  |  |  |  |  |  |  |  |  |  |  |  |  |  |
|  | Urban | **4.6** | 3.7 | 5.6 | **(0.9)** | 0 | 2.2 | **1.8** | 1 | 2.5 | **0.7** | 0 | 1.6 | **1.9** | 1.2 | 2.7 | **5.2** | 2.7 | 7.7 | **11.9** | 7.4 | 16.4 |
|  | Rural | **4.8** | 4 | 5.6 | **(1.6)** | 0 | 4.6 | **1.9** | 1.5 | 2.3 | **0.5** | 0 | 1.2 | **5.2** | 3.6 | 6.8 | **16.4** | 13.5 | 19.2 | **15.3** | 12.7 | 17.9 |
| **Education** | |  |  |  |  |  |  |  |  |  |  |  |  |  |  |  |  |  |  |  |  |  |
|  | Completed primary or less | **5** | 4.2 | 5.9 | **(1.7)** | 0 | 5 | **1.9** | 1.6 | 2.3 | **0.1** | 0 | 0.2 | **4.9** | 3.3 | 6.5 | **14** | 11.4 | 16.7 | **14.9** | 12.4 | 17.4 |
|  | Secondary or more | **4.4** | 3.5 | 5.3 | **(0.9)** | 0 | 2 | **1.7** | 1 | 2.4 | **1.3** | 0 | 2.7 | **2.3** | 1.4 | 3.2 | **11.1** | 7.5 | 14.7 | **13.5** | 8.4 | 18.5 |
| *Age, parity, marital status and contraceptive intention were measured at the end of the episode of contraceptive use; wealth, education and residence were measured at the time of the survey interview. (-) Not reported because there were fewer than 125 episodes of use of that method. Failure rates were calculated using the single-decrement approach. Values in parentheses indicate calculations were based on 125–249 episodes of use for that method. | | | | | | | | | | | | | | | | | | | | | | |
|  |  |  |  | pill-age RR: | pill weight | injection-age RR: | injection weight | male condom-age RR: | male condom weight | periodic abstinence-age RR: | periodic abstinence weight | withdrawal-age RR: | withdrawal weight |  |  |  |  |  |  |  |  |  |
|  | significant difference (no overlap in 95%CI between categories) | **Age RR (weighted average):** | **2.2** | 1.83784 | 5.51% | 2.38462 | 49.18% | 1.5 | 7.15% | 1.81553 | 1.56% | 1.36923 | 4.18% |  |  |  |  |  |  |  |  |  |

## Table A4.2 Proportional incremental change in Initiation_ByMethod rate (r_init1_age ~ Initiation_ByAge in ResourceFile_Contraception.xlsx) for each age in years of the woman (this is calculated for each age relative to the average initiation rate across all ages according to the best fitting model = 0.34196)

| age | r_init1_fracpoly | r_init1_age |
| --- | --- | --- |
| 15 | 0.4545 | 0.329 |
| 16 | 0.4187 | 0.225 |
| 17 | 0.3923 | 0.147 |
| 18 | 0.3726 | 0.090 |
| 19 | 0.3580 | 0.047 |
| 20 | 0.3472 | 0.015 |
| 21 | 0.3391 | -0.008 |
| 22 | 0.3333 | -0.025 |
| 23 | 0.3291 | -0.038 |
| 24 | 0.3261 | -0.046 |
| 25 | 0.3242 | -0.052 |
| 26 | 0.3231 | -0.055 |
| 27 | 0.3225 | -0.057 |
| 28 | 0.3224 | -0.057 |
| 29 | 0.3227 | -0.056 |
| 30 | 0.3232 | -0.055 |
| 31 | 0.3240 | -0.053 |
| 32 | 0.3249 | -0.050 |
| 33 | 0.3259 | -0.047 |
| 34 | 0.3271 | -0.044 |
| 35 | 0.3283 | -0.040 |
| 36 | 0.3295 | -0.036 |
| 37 | 0.3308 | -0.033 |
| 38 | 0.3321 | -0.029 |
| 39 | 0.3334 | -0.025 |
| 40 | 0.3348 | -0.021 |
| 41 | 0.3361 | -0.017 |
| 42 | 0.3373 | -0.014 |
| 43 | 0.3386 | -0.010 |
| 44 | 0.3398 | -0.006 |
| 45 | 0.3411 | -0.003 |
| 46 | 0.3423 | 0.001 |
| 47 | 0.3434 | 0.004 |
| 48 | 0.3446 | 0.008 |
| 49 | 0.3457 | 0.011 |
| Average: | 0.34196 |  |

## Figure A4.1 Contraception Initiation rate (all methods combined) by woman’s age in years, best fitting model is r_init1_fracpoly (blue line)


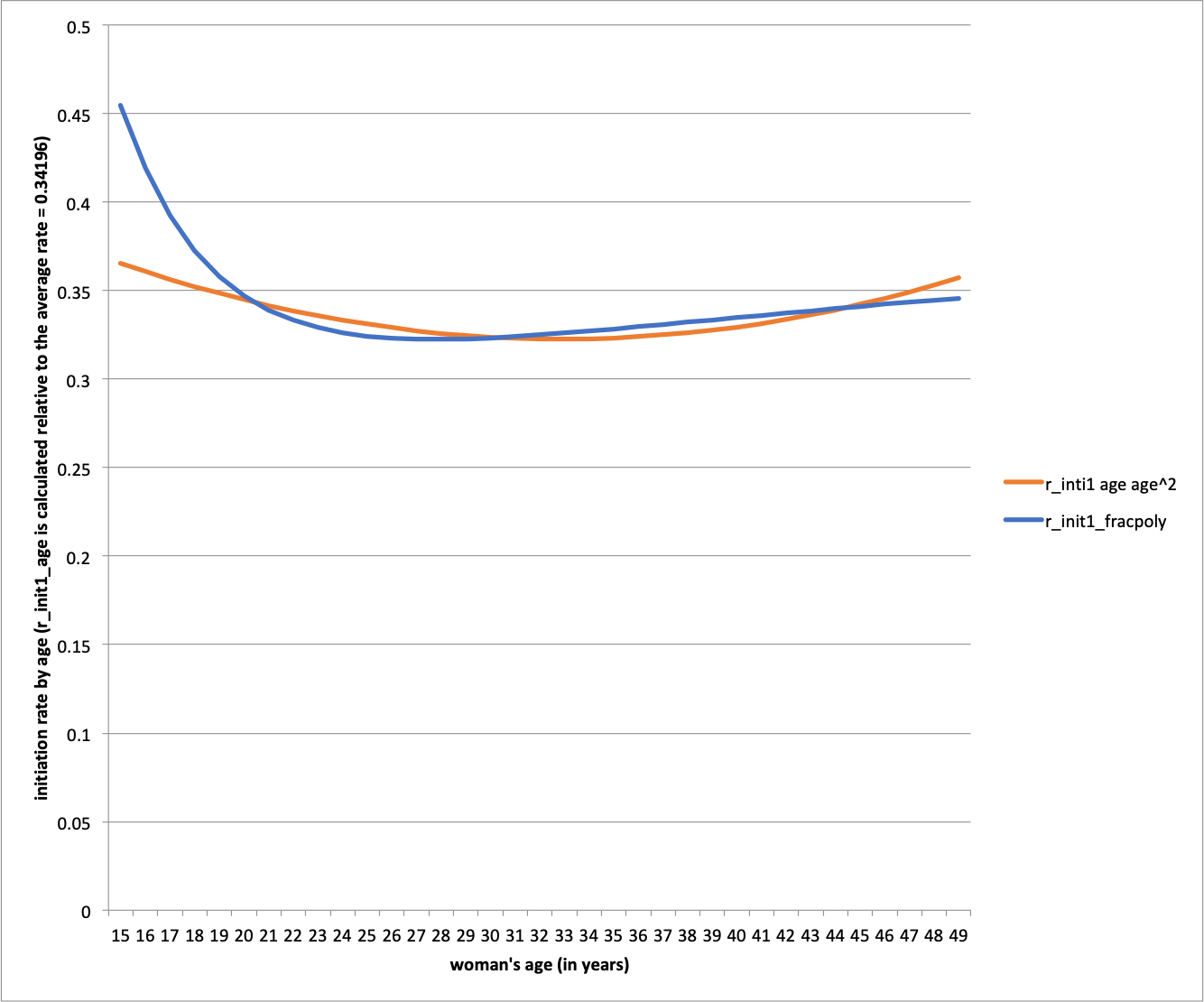


## Table A4.3 Proportional incremental change in Discontinuation_ByMethod rate (r_discont_age ~ Discontinuation_ByAge in ResourceFile_Contraception.xlsx) for each age in years of the woman (this is calculated for each age relative to the average initiation rate across all ages according to the best fitting model = 0.05635)

| age | r_discontinue_fracpoly | r_discont_age |
| --- | --- | --- |
| 15 | 0.0067 | -0.882 |
| 16 | 0.0209 | -0.629 |
| 17 | 0.0316 | -0.439 |
| 18 | 0.0397 | -0.295 |
| 19 | 0.0458 | -0.185 |
| 20 | 0.0505 | -0.102 |
| 21 | 0.0541 | -0.038 |
| 22 | 0.0569 | 0.011 |
| 23 | 0.0589 | 0.048 |
| 24 | 0.0605 | 0.075 |
| 25 | 0.0616 | 0.096 |
| 26 | 0.0625 | 0.110 |
| 27 | 0.0630 | 0.121 |
| 28 | 0.0634 | 0.127 |
| 29 | 0.0636 | 0.131 |
| 30 | 0.0637 | 0.133 |
| 31 | 0.0637 | 0.132 |
| 32 | 0.0636 | 0.131 |
| 33 | 0.0635 | 0.128 |
| 34 | 0.0633 | 0.124 |
| 35 | 0.0630 | 0.120 |
| 36 | 0.0627 | 0.115 |
| 37 | 0.0624 | 0.110 |
| 38 | 0.0621 | 0.104 |
| 39 | 0.0618 | 0.099 |
| 40 | 0.0615 | 0.093 |
| 41 | 0.0611 | 0.087 |
| 42 | 0.0608 | 0.080 |
| 43 | 0.0604 | 0.074 |
| 44 | 0.0601 | 0.068 |
| 45 | 0.0598 | 0.062 |
| 46 | 0.0594 | 0.056 |
| 47 | 0.0591 | 0.050 |
| 48 | 0.0588 | 0.044 |
| 49 | 0.0584 | 0.039 |
| Average: | 0.05625 |  |

## Figure A4.2 Contraception Discontinuation rate (all methods combined) by woman’s age in years, best fitting model is r_discont_fracpoly (blue line)


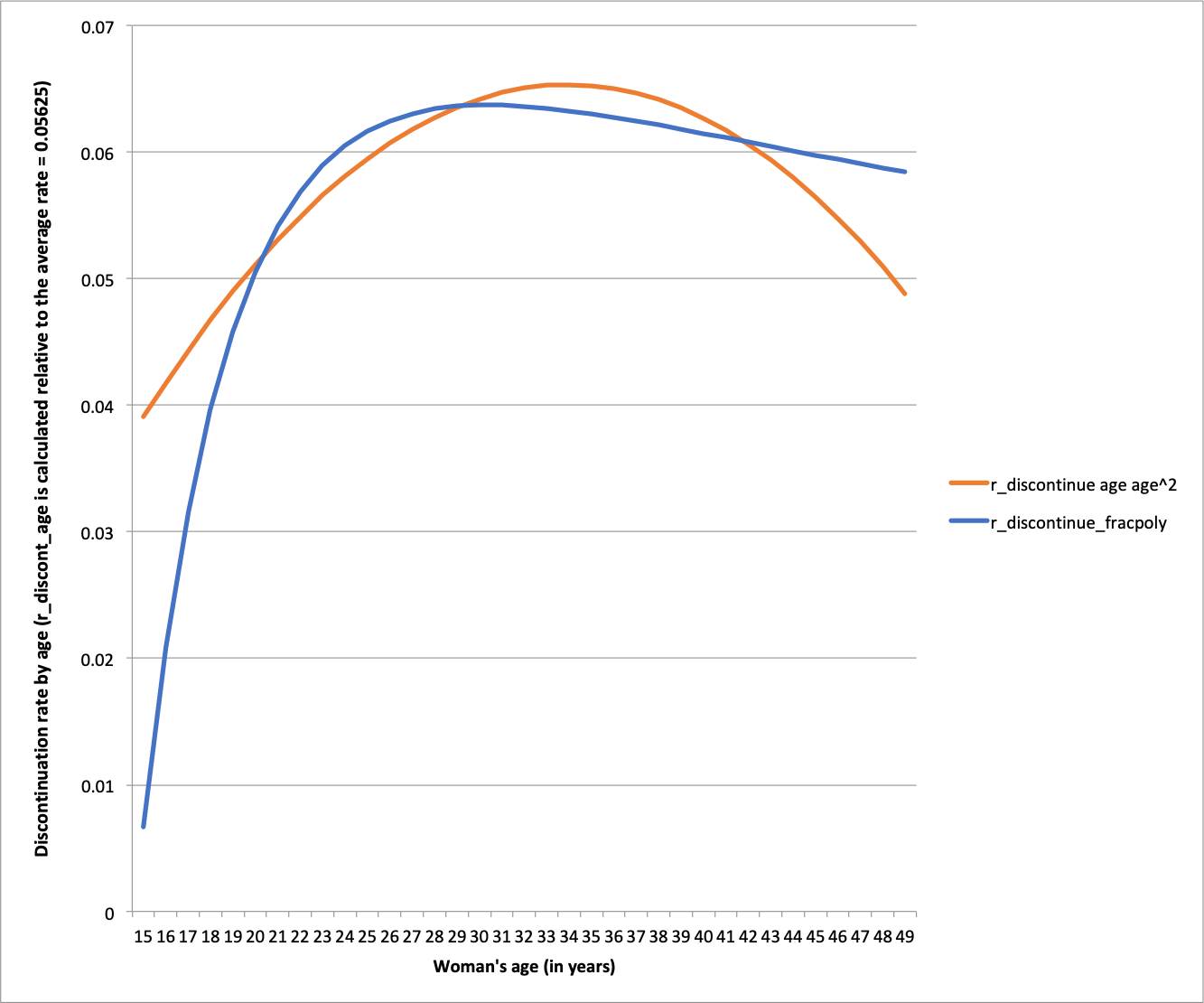


# Appendix 5: Contraception interventions

Table A5.1 shows contraception intervention parameters, (a) Interventions_Pop, the proportional increases in (multiplication of) Initiation_ByMethod by contraception method that represent Population-scope campaigns to increase contraception use (Pop); and (b) Interventions_PPFP, the proportional increases in (multiplication of) Initiation_AfterBirth that represent Post-partum family planning (PPFP). These multipliers are assumed to be reflective of the relative popularity of the different methods of contraception in Malawi – we have calibrated them to meet the expected changes in the percentage of women using each method from our 2010 baseline to 2020 as a result of the Malawi Costed Implementation Plan For Family Planning 2016-2020 (CIP), estimated on pages 37-38 (Figure 31) of the CIP report^4^. The 2010 baseline in the CIP report (Figure 31) is similar to that produced by our model. We then start Pop and PPFP interventions in 2016 as per the CIP and calibrate to expected percentages using each contraceptive method in 2020 (Figure 31 of CIP).

We assume both the Pop and PPFP interventions will only target increase in uptake of modern methods of contraception (so not periodic abstinence, withdrawal and other traditional whose initiation rates remain unchanged). We also assume no change in discontinuation rates given reasons for discontinuation are commonly due to wanting to become pregnant, wanting to switch to another method, or side-effects of contraception, all of which are unlikely to be affected by the demand creation Pop and PPFP interventions.

These increases are in addition to the expected secular increases in contraception use that mirror expected fertility decline and slowing population growth in Malawi according to the UN World Population Prospects^5^ as explained in Appendix 1.

The Pop intervention costs per population size of 2016 are estimated via the costs for the ‘Demand creation’ thematic area activities of the CIP^4^. This is the latest available costed implementation plan, and costed demand creation at a total of 6.6 billion Malawi Kwacha (MWK) over the 5 year period 2016-2020. We therefore assume an annual costs of the Pop intervention of 1.3 billion Malawi Kwacha (MWK) in 2016. Demand creation activities include information, education and communication outreach and social behaviour change communication campaigns including use of mass media, peer educators, community engagement, and engagement of key stakeholder groups including youth in and out of school, and religious, traditional and community leaders.

The PPFP intervention costs per population size of 2016 are estimated via the costs of the ‘Service delivery and access’ thematic area activities of the CIP, which costed service delivery and access at 7.3 billion Malawi Kwacha (MWK) over the 5 year period 2016-2020^4^. These costs cover 10 activities of which one (SDA.9) is integration of contraception (family planning) into a variety of services as well as postpartum care. We therefore conservatively assume the annual costs of integration of the provision of contraception into post-partum care (PPFP) are one tenth of the total costs of service delivery and access, which is 146 million Malawi Kwacha (MWK) in 2016.

The costs used in the CIP are given in 2015 MWK and we inflate them to 2021 MWK (inflation of 81%) via consumer price index data^6^ (Table A5.1).

These annual national costs for 2016 are assumed to reflect the size of the population of women and men of reproductive age (15-49) covered by these interventions, hence we adjust them for each year simulated with interventions accordingly by the size of the population, with costs increasing proportionally to the population increase.

## Table A5.1 Contraception intervention parameters (a Interventions_Pop, b Interventions_PPFP in ResourceFile_Contraception.xlsx) and costs

| **Contraception method** | **(a) Pop intervention multiplier of Initiation_ByMethod** | **(b) PPFP intervention multiplier of Initiation_AfterBirth** |
| --- | --- | --- |
| pill | 2.5 | 2.5 |
| IUD | 11 | 11 |
| injection | 1.6 | 1.6 |
| implant | 8 | 8 |
| male condom | 2.6 | 2.6 |
| female sterilization | 2 | 2 |
| other modern | 5 | 5 |
| periodic abstinence | 1 | 1 |
| withdrawal | 1 | 1 |
| other_traditional | 1 | 1 |
| **Annual intervention costs per population size of 2016^4^ (inflated to 2021 MWK**^a^**)** | **2.4 billion MWK** | **264 million MWK** |

^a^ Pop intervention implementation cost of 1.3 billion MWK and PPFP intervention implementation cost of 146 million MWK inflated from 2015 MWK using consumer price index (CPI) data^6^; CPI in 2015 = 250.6, CPI in 2020 = 454.4, an increase of (454.4-250.6)/250.6 ~ 81%.

# Appendix 6: Contraception use and pregnancies – numbers of women

## Figure A6.1 Model results without and with Pop and PPFP interventions

**(A)** Total contraception use per month,

**(B)** Total number of pregnancies per year,

**(C)** Total contraception use by method per month;

**(Left)** without Pop and PPFP interventions, **(Right)** with Pop and PPFP interventions since 2023

**dotted vertical line** shows the start of the interventions, year 2023


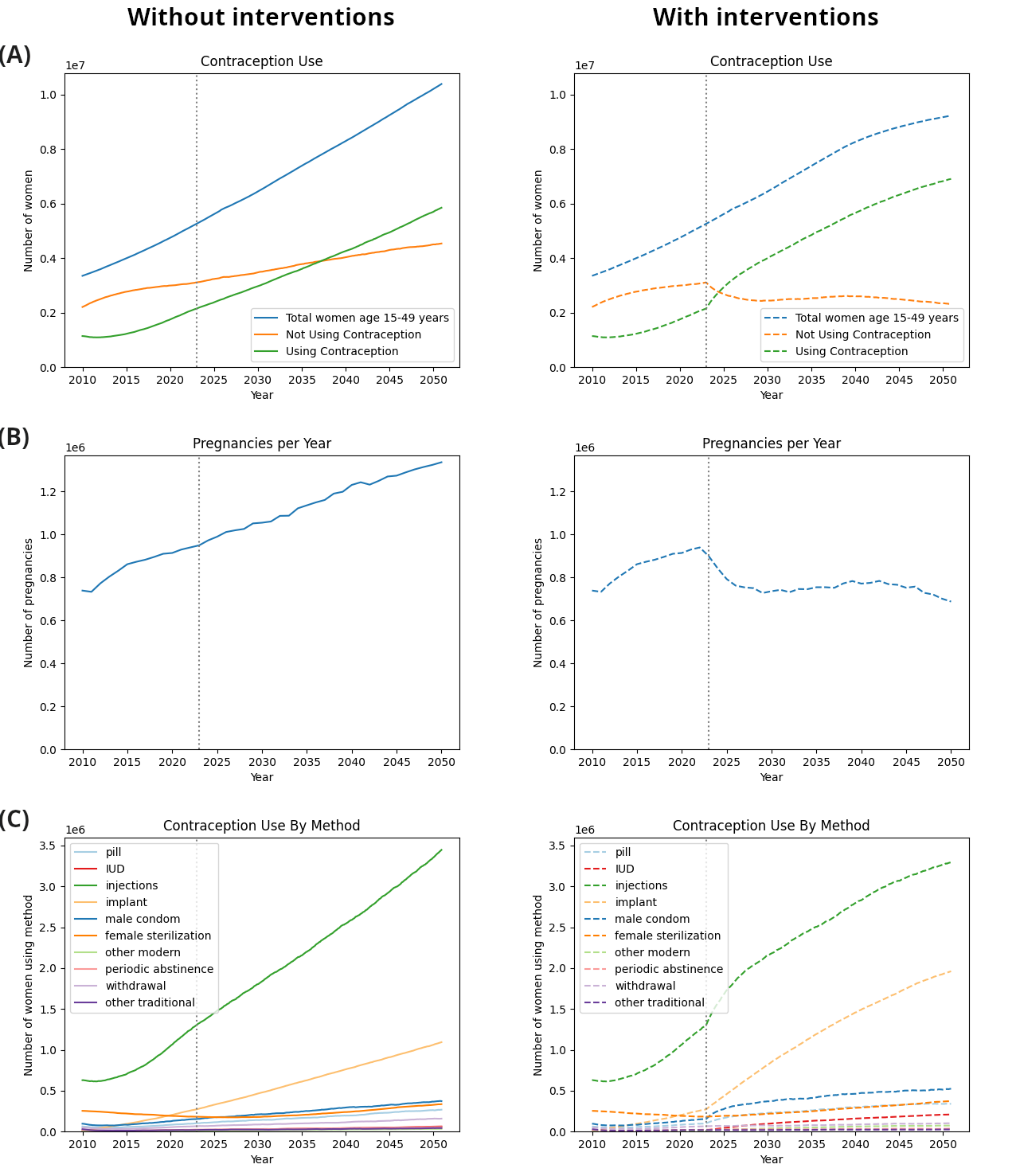


# References

1. Coviello V, Boggess M. Cumulative incidence estimation in the presence of competing risks. *Stata Journal* 2004; **4**(2): 103-12.

2. Choudhury JB. Non-parametric confidence interval estimation for competing risks analysis: application to contraceptive data. *Stat Med* 2002; **21**(8): 1129-44.

3. Polis CB, Bradley SEK, Bankole A, Onda T, Croft T, Singh S. Contraceptive Failure Rates in the Developing World: An Analysis of Demographic and Health Survey Data in 43 Countries ( <http://www.guttmacher.org/report/contraceptive-failure-rates-in-developing-world> accessed 25th November 2019). New York: Guttmacher Institute, 2016.

4. Government of Malawi. Malawi Costed Implementation Plan For Family Planning, 2016–2020. Lilongwe: Government of Malawi, 2015.

5. United Nations Department of Economic and Social Affairs Population Division. World Population Prospects 2019, Volume I: Comprehensive Tables (ST/ESA/SER.A/426). , 2019.

6. World Bank. Consumer Price Index (2010=100) – Malawi, 2015–2020. <https://data.worldbank.org/indicator/FP.CPI.TOTL?end=2020&locations=MW&start=2015&view=chart> (accessed 10th May 2023). 2023.
